# Small-molecule activators of the *Staphylococcus aureus* ClpC/ClpP AAA+ protease

**DOI:** 10.1016/j.isci.2026.117738

**Published:** 2026-09-22

**Authors:** Timo Jenne, Vsevolod Viliuga, Ulrike Uhrig, Britta Jehle, Merlin Schwan, Jürgen Kopp, Dirk Flemming, Elisabeth Seebach, Irmgard Sinning, Bernd Bukau, Axel Mogk

**Affiliations:** 1 Center for Molecular Biology of Heidelberg University (ZMBH), DKFZ-ZMBH Alliance, Im Neuenheimer Feld 345, 69120 Heidelberg, Germany; 2 Department of Biochemistry and Biophysics at Stockholm University and Science for Life Laboratory, Stockholm, Sweden; 3 The European Molecular Biology Laboratory, EMBL, Meyerhofstraße 1, 69120 Heidelberg, Germany; 4 Heidelberg University Biochemistry Center (BZH), Im Neuenheimer Feld 328, 69120 Heidelberg, Germany; 5 Department of Infectious Diseases, Medical Microbiology and Hygiene, Medical Faculty Heidelberg, Heidelberg University, Heidelberg, Germany; 6 University Hospital Heidelberg, Heidelberg, Germany

**Keywords:** protein degradation, AAA protein, Hsp100, protease, chaperone, bacteria, ClpP, protein quality control, SAR, MoA

## Abstract

The central AAA+ ClpC/ClpP protease in Gram-positive bacteria is crucial for virulence and stress resistance and has been recognized as drug target. Natural cyclic peptides deregulate the essential *Mycobacterium tuberculosis* ClpC1 and cause cell death. Similarly, overactivated mutants of the non-essential *Staphylococcus aureus* ClpC homolog cause uncontrolled proteolysis and severe toxicity *in vivo*. However, no chemical modulators of *S. aureus* ClpC have been described. Here, using a biochemical high-throughput screen, we identify eight distinct *bona fide* small molecules that stimulate ClpC ATPase and proteolytic activity *in vitro*. Structural, computational, and mutational analyses define two ligandable regulatory sites within the ClpC N-terminal domain (NTD) as compound targets: a conserved hydrophobic groove and an allosteric pArg1 pocket, both engaged in substrate recognition. These findings establish *S. aureus* ClpC as chemically targetable and provide mechanistic insight into its regulatory architecture, enabling future development and optimization of chemical probes to deregulate AAA+ protease control.

## Highlights

• Small molecules enabling autonomous *S. aureus* ClpC/ClpP protease activity
• Compounds target ClpC and increase its ATPase activity
• Compounds bind to two distinct sites of the ClpC NTD
• pArg1-pocket of NTD identified as a new druggable site for small molecules

## Graphical abstract

**Figure:**
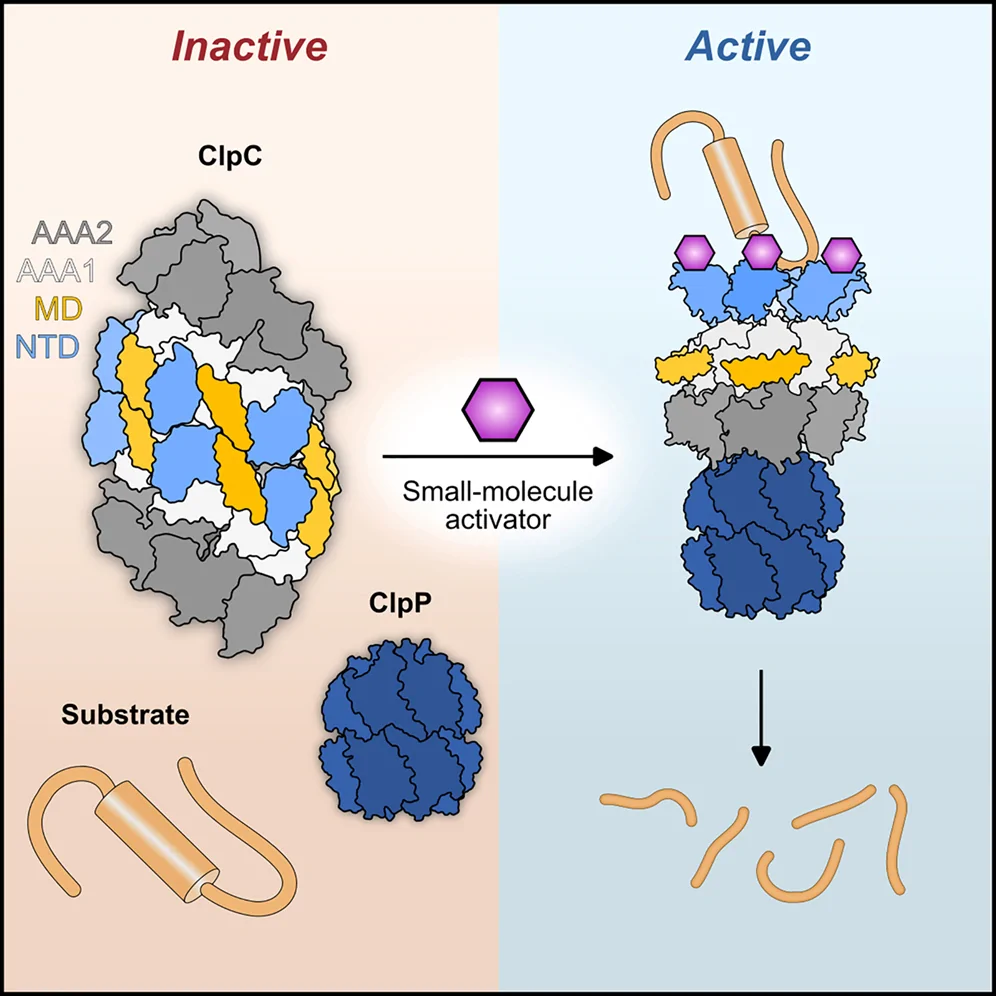

## Teaser

molecular microbiology; structural biology

## Introduction

Recently, there has been a significant increase in hospitalized patients with difficult-to-treat infections caused by drug-resistant bacteria, a trend most pronounced for the pathogen *Staphylococcus aureus*.^1^ While strains such as totally drug-resistant *Mycobacterium tuberculosis* (TDR-TB) evolved^2^ and methicillin-resistant *S. aureus* (MRSA) strains emerge at high frequency,^3^ the number of new antibiotics developed over the last 50 years is very small.^4^ Traditional antibiotic targets such as protein biosynthesis and DNA replication have been largely exhausted,^5^ demanding the identification of new antibacterial targets for antibiotic development.

One promising new drug target is the central ClpC/ClpP protease in Gram-positive bacteria, a functional equivalent of the eukaryotic 26S proteasome.^6^ The ClpC/ClpP proteolytic machinery consists of two major components, the AAA+ (ATPase associated with diverse cellular activities) unfoldase ClpC and the barrel-shaped serine peptidase ClpP. ClpC consists of two ATPase domains (AAA1, AAA2), an N-terminal domain (NTD), and a coiled-coil M-domain (MD). In its active state, ClpC assembles into a hexameric ring and threads substrates in an ATP-dependent manner through its central translocation channel directly into the associated proteolytic chamber of ClpP.^7^ To prevent unspecific unfolding and degradation events, ClpC activity and substrate specificity are tightly regulated. In its basal state, ClpC forms an inactive resting state composed of two half-spirals.^8^^,^^9^ In this resting state, ClpC exhibits low ATPase activity and cannot associate with ClpP. This assembly state is stabilized by head-to-head-interacting MDs and by the binding of the NTD to MDs and the AAA1 domain. ClpC activation involves hexamer formation mediated by various adapter proteins that bind to the NTD (MdfA and McsB) or both MD and NTD (MecA).^10^^,^^11^^,^^12^ Alternatively, polypeptides harboring a phosphoarginine (pArg) degron tag created by the McsA/McsB arginine kinase are specifically recognized by two conserved binding pockets (pArg1/pArg2) at the NTD, also triggering ClpC activation.^9^^,^^13^^,^^14^ Importantly, MecA and McsB target themselves for degradation in the absence of substrate, forming a negative feedback loop that restricts ClpC activity to the presence of substrates.^12^^,^^15^

ClpC/ClpP is crucial for stress resistance, virulence, and biofilm formation in various pathogens such as *S. aureus*, *Listeria monocytogenes,* and *Streptococcus pneumoniae*.^16^^,^^17^^,^^18^^,^^19^^,^^20^^,^^21^ In mycobacteria, the homologous ClpC1/ClpP1P2 protease is even essential for cell viability.^22^^,^^23^ Multiple screening campaigns identified *M. tuberculosis* ClpC1 and also ClpP1P2 as a potential drug target.^24^^,^^25^^,^^26^^,^^27^^,^^28^^,^^29^ Additionally, deregulating ClpP from *S. aureus*, *Escherichia coli, Neisseria* sp., and *Bacillus subtilis* has been shown to represent a viable strategy for antibiotic development.^30^^,^^31^^,^^32^^,^^33^

One class of these antibiotics targeting ClpP are acyldepsipeptides (ADEPs).^30^ ADEPs deregulate ClpP by mimicking AAA+ partner docking by binding to ClpP H-sites, triggering formation of the active proteolytic site and opening of the ClpP entry pores.^34^ This enables ClpP to autonomously degrade loosely folded proteins, including nascent polypeptides, causing toxicity.^30^^,^^35^ Furthermore, ADEPs disrupt AAA+ partner-peptidase cooperation, thereby stabilizing native substrates, causing lethality in *M. tuberculosis*.^29^^,^^30^^,^^35^ ADEPs have been shown to be highly effective and even eliminate hard-to-kill persister cells.^36^

Molecules that target ClpC1 encompass several highly toxic natural cyclic peptides, including cyclomarin A (CymA), lassomycin, rufomycin, and ecumicin.^24^^,^^25^^,^^26^^,^^28^ All these molecules bind to a conserved hydrophobic groove of the ClpC1 NTD, which functions as substrate-binding site.^37^^,^^38^^,^^39^^,^^40^ Notably, the effects of cyclic peptides on ClpC1/ClpP1P2 activity vary. Peptides typically increase ClpC1 ATPase activity, yet can have both stimulatory and inhibitory impacts on proteolytic activities.^24^^,^^25^^,^^26^^,^^41^^,^^42^ Similarly, cyclic peptides differ substantially in their impact on the *M. tuberculosis* proteome, suggesting diverse modes of ClpC1 deregulation.^43^ Newly developed CymA-based degraders (BacPROTACs) enable targeted degradation of specific proteins of interest or even ClpC1 itself, representing alternative strategies to disturb protein homeostasis via ClpC1/ClpP1P2.^44^^,^^45^

Although ClpC homologs share high sequence homology, the previously described cyclic peptides are highly specific to mycobacteria^24^^,^^25^^,^^26^ and do not target, e.g., *S. aureus* ClpC. Yet, transplanting the NTD of *M. tuberculosis* ClpC1 to *S. aureus* ClpC leads to its sensitization toward CymA, and the fusion construct creates toxicity by uncontrolled proteolysis when expressed in *E. coli* in the presence of CymA.^41^ This suggests that an induced, toxic deregulation of *S. aureus* ClpC/ClpP is possible, although ClpC and ClpP are not essential.^17^ Accordingly, expression of deregulated *S. aureus* ClpC mutants, which exhibit autonomous, uncontrolled activities, is highly harmful to *E. coli* cells.^8^^,^^9^ We therefore sought to identify small molecules that trigger persistent *S. aureus* ClpC/ClpP activation to disrupt protein homeostasis and conducted a high-throughput screen. We present the first examples of small-molecule activation of the *S. aureus* ClpC AAA+ unfoldase *in vitro*. We identify eight *bona fide* compounds targeting two chemically ligandable regulatory sites of the NTD—the hydrophobic groove and the pArg1 pocket. Additionally, we clarified the roles of these compounds in allosteric activation of ClpC through co-crystallography, computational approaches, and biochemically by site-directed mutagenesis. The identified compounds impair *S. aureus* cell growth, however, in a ClpC/ClpP-independent manner, and are therefore in need of further optimization. Together, our findings provide a foundation for the development of new tools for protease deregulation as a potential antibiotic strategy.

## Results

### High-throughput screen identifies small-molecule activators of *S. aureus* ClpC

To identify compounds that activate the *S. aureus* ClpC/ClpP protease, we conducted a high-throughput screen using a chemically diverse library of approximately 110,000 small molecules (Figure S1). Compounds were screened at a single concentration (40 μM) for increasing ClpC/ClpP proteolytic activity, which was measured using fluorescently labeled FITC-casein as model substrate. Degradation of FITC-casein causes an increase in fluorescence intensity, which was measured in an endpoint experiment after 60 min of incubation in the absence and presence of compounds (Figure 1A). On each assay plate, negative controls (ClpC and ClpP only) and positive controls (ClpC, ClpP and the adapter protein MecA) were measured. This enabled normalization for each plate and monitoring of assay performance with Z′-factors. Assay quality, as assessed by the Z′-factor, ranged between 0 and 0.6 across screening plates, indicating rather low robustness. This might be explained by the fact that the adaptor MecA (= positive control), being itself a substrate, is degraded over time and thus the measured activity values for the positive controls show a high variance. Therefore, 13 out of 337 assay plates were not taken into account as the Z′-values were below zero. However, even with the low Z′-prime values, there was sufficient separation between the signal of positive and negative controls which enabled hit identification. The cutoff value was selected after analyzing the distribution of measured activity values for positive and negative controls and screened compounds (Figure S2). It corresponds roughly to the mean activation value of all positive control wells minus one time the standard deviation. This yielded approximately 900 preliminary hits, which is a relatively high hit rate. To prioritize promising scaffolds and reduce redundancy, hit compounds were analyzed using chemical structure clustering. Only compound families with multiple active analogs were selected for follow-up testing, while singletons (more than 670 compounds) were deprioritized. This selection process for filtering a big hitlist is reasonable, as the screening library is set up to have compound families, and similar compounds should exhibit similar biological effects and be identified as hit compounds. Thus, representative compounds from prioritized clusters were subsequently selected. Taking into account availabilities, 57 compounds were bought from the vendors from which the compounds were originally acquired for further biochemical and mechanistic characterization (Figure S1).

**Figure 1:**
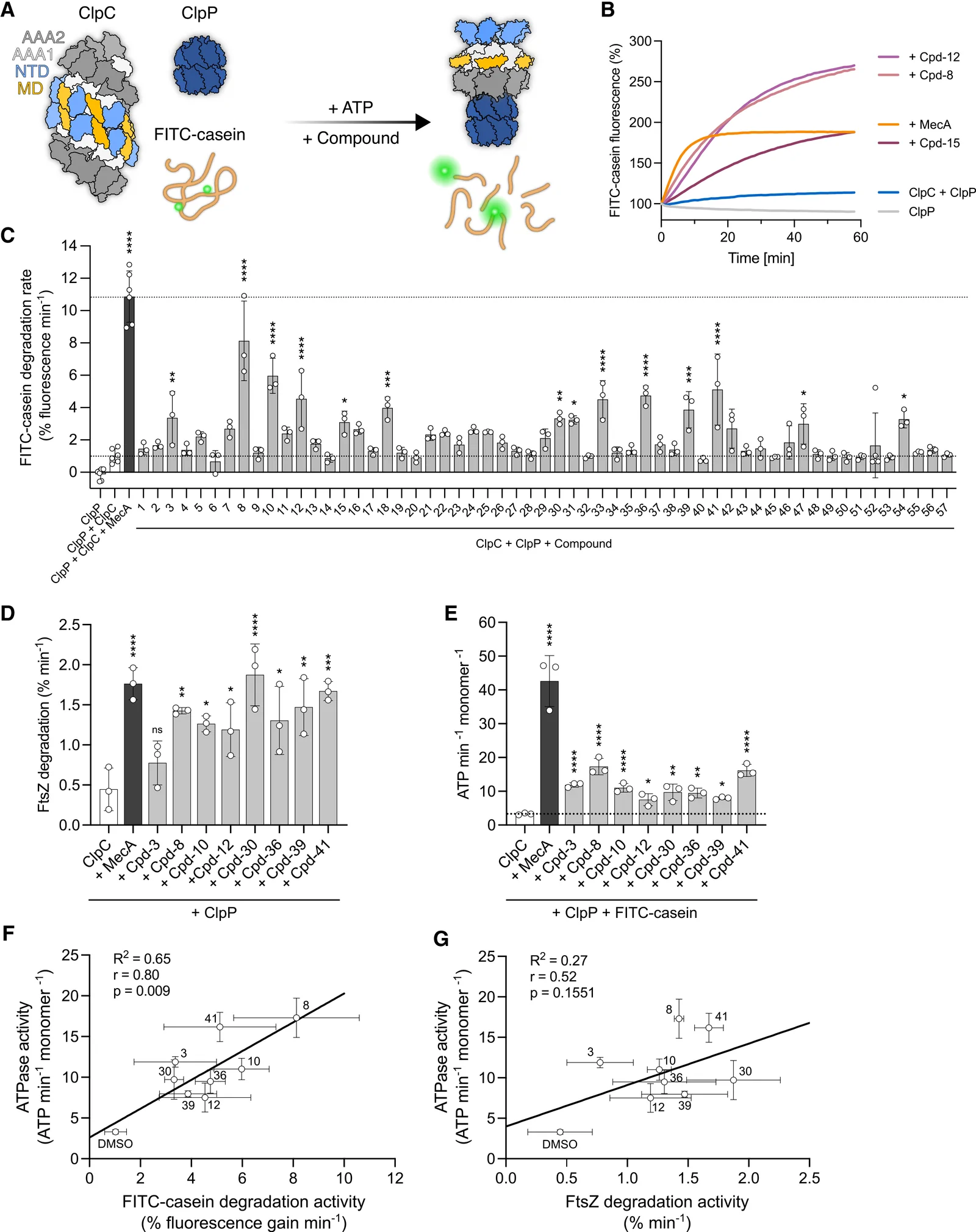
Small-molecule activators identified in a high-throughput screen activate *S. aureus* ClpC *in vitro* (A) Model illustrating ClpC activity control and the functional principle of the FITC-casein degradation assay used for high-throughput screening. In its resting state, ClpC is inactive and unable to engage ClpP. Compound-induced activation promotes hexamer assembly, allowing formation of an active ClpC/ClpP protease that degrades FITC-casein, monitored as fluorescence dequenching. (B) Representative curves of proteolytic activities of ClpP and ClpC + ClpP ± MecA or selected compounds toward FITC-casein. Initial FITC-casein fluorescence was set to 100%. (C) FITC-casein degradation activities were determined in the presence of ClpP and ClpC + ClpP ± MecA or respective compounds (50 μM), *n* ≥ 3. (D) FtsZ degradation activities were determined in the presence of ClpP ± MecA or respective compounds (50 μM), *n* = 3. (E) ATPase activities of ClpC were determined in the presence of ClpP and FITC-casein ± MecA or respective compounds (50 μM), *n* = 3. (F and G) Correlation of ATPase with FITC-casein (F) or FtsZ (G) degradation activities (shown in C–E) in presence of the tested compounds was calculated using two-tailed Pearson correlation tests. DMSO-treated ClpC/ClpP served as negative control. All data are represented as mean ± SD. Statistical significance was determined by one-way ANOVA with Dunnett’s post-hoc test (C, D, and E). Positive controls were assessed separately. ns, not significant; ∗*p* < 0.05, ∗∗*p* < 0.01, ∗∗∗*p* < 0.001, ∗∗∗∗*p* < 0.0001. See also Figures S1–S4.

### Biochemical validation and characterization of primary hits

Next, the 57 primary hits (Figure S3) were tested in a time-resolved FITC-casein degradation assay for better quantification of their stimulatory impact but also to exclude false-positive candidates (Figures 1B, 1C, and S4A). Approximately 50% of the compounds failed to reproducibly stimulate ClpC/ClpP activity and were classified as false positives. The remaining compounds induced robust FITC-casein degradation, as shown by representative kinetic traces in Figure 1B and calculated degradation rates (% fluorescence increase min^−1^) in Figure 1C. Basal ClpC/ClpP activity was low (1.03% fluorescence increase min^−1^) and increased strongly upon addition of the adaptor protein MecA, which served as a positive control and exhibited the highest initial degradation rate (10.87% fluorescence increase min^−1^). Degradation kinetics of the positive control reached an early plateau as MecA targets itself for degradation by ClpC/ClpP (Figure 1B). In contrast, the small-molecule activators supported sustained protease activity and resulted in higher total FITC-casein degradation after 60 min, indicating that they were not consumed during the reaction (Figure S4A). The most significant increase was observed with compound 8 (Cpd-8), which enhanced ClpC/ClpP activity by a factor of 7.9 (8.13% fluorescence increase min^−1^), approaching the degradation rates mediated by MecA (10.6-fold increase). We selected 12 compounds that led to significant enhancement of ClpC/ClpP proteolytic activity using kinetics (% FITC-casein fluorescence/min, Figure 1C) and total efficiency (% FITC-casein fluorescence after 60 min, Figure S4A) of FITC-casein degradation as criteria. Initial analysis of Cpd-33 did not yield significant stimulation and was therefore not further investigated. We determined the efficiencies of these 12 compounds using dose-response curves. The compounds' potencies were in the low-to moderate micromolar range, with compounds 8, 10, and 41 showing the lowest EC50 values (Cpd-8: 12 μM; Cpd-10: 4.4 μM; Cpd-41: 8 μM) (Figures S4B and S4C). Building on these findings, we excluded Cpd-15, Cpd-18, Cpd-31, and Cpd-54 from further analyses and focused on the remaining eight compounds (Cpd-3, Cpd-8, Cpd-10, Cpd-12, Cpd-30, Cpd-36, Cpd-39, and Cpd-41). Lastly, we conducted a ClpP counter-assay to rule out ClpC-independent FITC-casein degradation by ClpP only in the presence of compounds. ClpP alone did not degrade FITC-casein, yet binding of ADEP1, which served as a positive control, caused substrate proteolysis (2.0% fluorescence increase min^−1^) (Figure S4D). None of the tested compounds enabled ClpP to degrade FITC-casein, suggesting that they target ClpC.

Next, we used the bacterial cell division protein FtsZ as an alternative, native substrate of ClpC/ClpP^46^ in an SDS-PAGE-based degradation assay. All eight compounds increased FtsZ degradation rates by 1.7- to 4.2-fold, with Cpd-30 showing the greatest effect and resulting in total FtsZ degradation up to 100% after 120 min (Figures 1D, S4E, and S4F). These results demonstrate that the compound efficacies are largely independent of the substrate. We also confirmed degradation of FITC-casein upon ClpC activation by SDS-PAGE for selected compounds (Cpd-8, Cpd-10, and Cpd-41), ensuring that the fluorescence-based degradation assay faithfully reports on FITC-casein proteolysis (Figure S4G).

To directly link enhanced proteolytic activity to ClpC activation, we measured the ATPase activity of ClpC in the presence of compounds with or without the addition of either ClpP, FITC-casein, or both (Figures 1E, S4H, and S4I). All compounds significantly stimulated ClpC ATPase activity (2.2- to 4.9-fold), but to a lesser extent than MecA-mediated activation (12.9-fold) (Figure 1E). The most pronounced effects were typically observed in the presence of both ClpP and FITC-casein (Figure S4H), with Cpd-8 and Cpd-41 inducing the highest ATPase activity (5.2- and 4.9-fold, respectively); however, the impact of ClpP and FITC-casein varied across compounds (Figures S4H and S4I). Importantly, we observed a correlation between ClpC ATPase activation and ClpC/ClpP proteolytic activities, indicating that compounds act by overriding ClpC activity control (Figures 1F and 1G). Surprisingly, Cpd-10 inhibited ClpC ATPase activity in the absence of ClpP and FITC-casein. This inhibition was countered by adding ClpP, whereas an activating effect was achieved by adding FITC-casein (Figures S4H and S4I). Collectively, these data demonstrate that the identified compounds activate ClpC independently of substrate identity or assay format. Together, these results identify a set of small-molecule activators that deregulate *S. aureus* ClpC, leading to increased ATP turnover and persistent ClpC/ClpP proteolytic activity.

### Compounds trigger ClpC conformational rearrangements that promote ClpP interaction

The increased activity state of ClpC indicates that the compounds influence its assembly, causing hexamer formation and subsequent association with ClpP. Thus, we used analytical size-exclusion chromatography (SEC) to observe ClpC association with ClpP. Here, we used ATPase-deficient ClpC-E280A/E618A (ClpC-DWB) to stabilize potential complexes with ClpP. ClpC-DWB eluted prior to a 670 kDa standard and was unable to form complexes with ClpP, indicating resting state formation (Figure 2A). The addition of MecA induced hexamer formation, enabling efficient protease complex formation as shown by the co-elution of ClpC-DWB, MecA, and ClpP prior to the ClpC resting state. Presence of Cpd-8, Cpd-10, or Cpd-41 also induced the formation of ClpC/ClpP assemblies, consistent with ClpC/ClpP activation. Complex formation was, however, less efficient, which could be explained by dissociation of the compounds during the SEC runs. Furthermore, compound-triggered ClpC/ClpP complexes were larger than the canonical ClpC/ClpP/MecA complex, eluting close to the void volume of the SEC column. To understand the structural organization of these compound-induced ClpC/ClpP assemblies, we analyzed the early-eluting SEC fractions by negative-stain electron microscopy (EM) (Figure 2B). ClpC/ClpP complexes appeared very large and highly heterogeneous, yet consisted of interacting ClpC hexamers interconnected by ClpP (Figures 2B and S5). Exemplarily, we obtained 2D class averages of some complexes in the presence of Cpd-8, which we suggest represent building blocks of larger assemblies (Figures 2B and S5A). The simplest element consisted of two ClpC hexamers interacting head-to-head, presumably through their coiled-coil middle domains (MDs), while being associated with ClpP. Such ring-dimer formation has recently been shown for the AAA+ disaggregase ClpL^47^^,^^48^ and for ClpP-bound *S. aureus* ΔN-ClpC, lacking its NTD, or partially in the presence of the substrate FITC-casein and phosphoarginine (pArg) acting as allosteric activator.^9^ Other building blocks consisted of four ClpC hexamers forming a tetrahedral assembly that associates with multiple ClpPs (Figure 2B). ClpC tetrahedrons have been shown to form in the presence of the toxic cyclic peptide Cyclomarin A,^41^^,^^42^ pArg-substrates,^9^^,^^44^ or derepressed ClpC mutants in the presence of substrate FITC-casein,^9^ respectively. In such a tetrahedral assembly, ClpC hexamers interact again via head-to-head interacting MDs. We also identified complexes comprising at least five ClpC hexamers, an assembly that has not been reported yet. Similar organizations of large ClpC/ClpP complexes were observed in the presence of Cpd-10 and Cpd-41 (Figures S5B and S5C).

**Figure 2:**
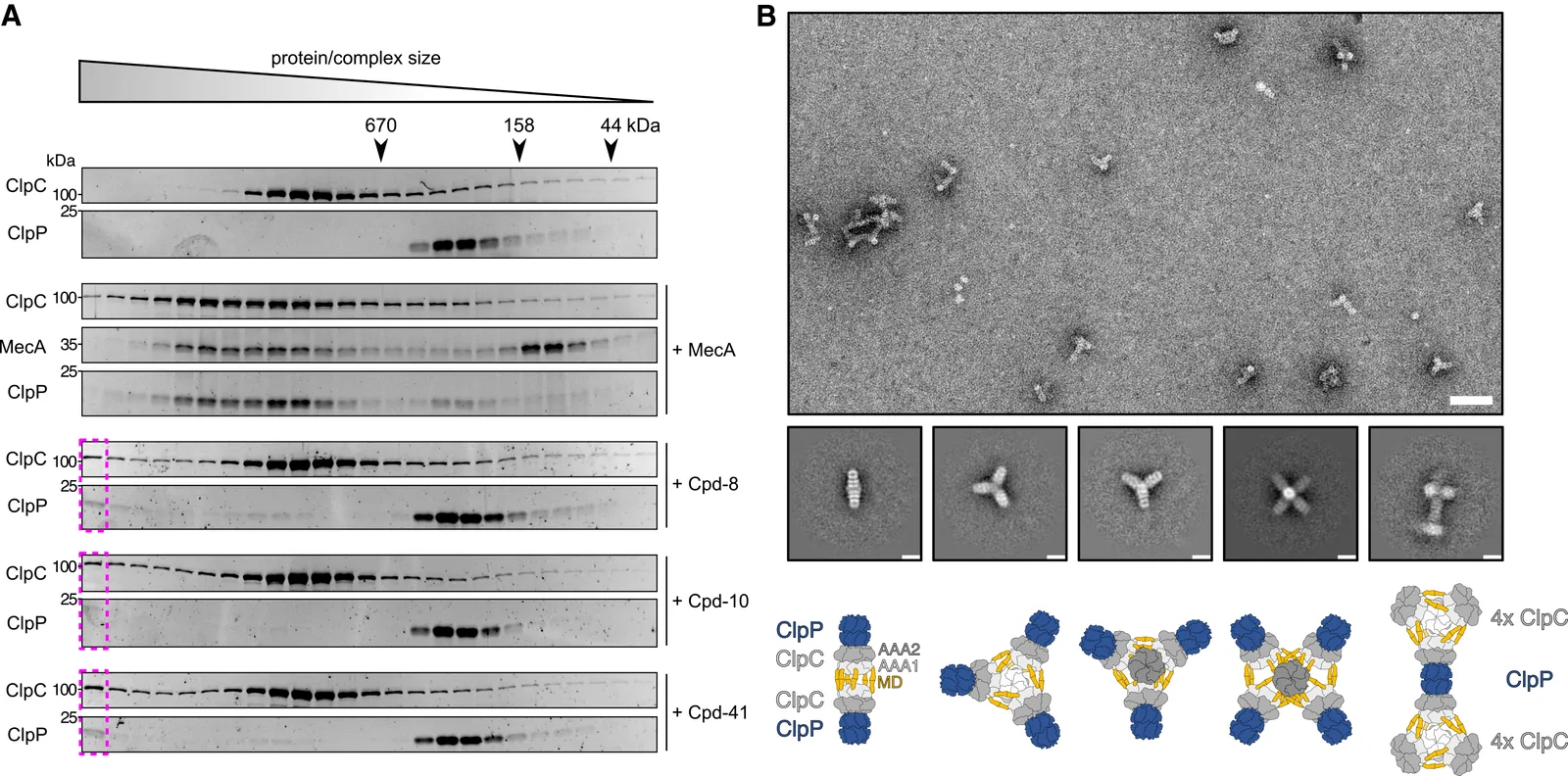
Compounds trigger conformational rearrangements that activate ClpC and promote ClpP interaction (A) Complex formation of ATPase-deficient ClpC-E280A/E618A (DWB) with ClpP and FITC-casein ± MecA or respective compounds (50 μM) was monitored by Superose 6 size-exclusion chromatography. Elution fractions were analyzed by SDS-PAGE and SyproRuby staining. (B) Negative-stain EM of ClpC-DWB/ClpP complexes formed in the presence of FITC-casein and Cpd-8 as isolated from SEC runs (see magenta boxes in A). Representative micrograph (scale bar, 50 nm) and 2D class averages (scale bars, 20 nm) reveal the formation of variable ClpC-DWB/ClpP complexes including rods, tetrahedrons, and other higher-order complexes composed of ClpC-DWB hexameric rings that interact head-to-head through coiled-coil M-domains. These complexes are connected through tetradecameric ClpP, which offers two docking sites for ClpC hexamers. The structural organizations of representative 2D classes are depicted as cartoons highlighting ClpP (dark blue) and ClpC domains (MD, orange; AAA1, white/light gray; AAA2, gray). See also Figure S5.

Taken together, the tested compounds induce ClpC hexamer formation and the subsequent development of large ClpC assemblies with remarkably high structural variability. These assemblies likely form via MD-MD interactions and have ClpC AAA2 rings accessible for ClpP docking. This, combined with ClpP’s ability to bind two ClpC hexamers, allows for the formation of extensive networks of ClpC assemblies interconnected by ClpP.

### The NTD serves as compound binding site

Next, we aimed to identify the compound binding sites to better understand their mechanisms of action (MoA). The NTD of Mtb ClpC1 is the target of all currently described toxic cyclic peptides, which deregulate ClpC1 and cause severe toxicity.^37^^,^^38^^,^^39^^,^^40^ Furthermore, the NTD has recently been identified as a central regulatory allosteric hub in ClpC activity control, stabilizing the resting state and integrating various input signals like adaptor proteins (e.g., MecA) or the pArg-degron tag for ClpC activation.^9^ Therefore, we first used a ClpC variant lacking its NTD (ΔN-ClpC) and measured its ATPase activity in the absence and presence of compounds (Figure 3A). None of the compounds stimulated ΔN-ClpC ATPase activity, suggesting that the NTD is the compound-binding site. Cpd-10 even inhibited ΔN-ClpC ATPase activity by a factor of 3.3, while the addition of FITC-casein and ClpP lessened this inhibitory effect. This inhibition, which we also observed in part for ClpC-WT (Figures S4H and S4I), suggests the existence of a secondary Cpd-10-binding site with lower affinity outside the NTD. Next, we evaluated whether compounds could enhance the proteolytic activity of ΔN-ClpC/ClpP. None of the compounds was able to increase proteolytic activity, supporting our assumption that all compounds bind to the NTD (Figure 3B). ΔN-ClpC-F436A, a constitutively activated ClpC variant, served as positive control documenting that FITC-casein degradation is possible without the NTD, which harbors a hydrophobic groove that binds unfolded proteins like casein.^9^^,^^49^ To further document compound binding to the NTD, we used a reciprocal approach and added an excess of isolated NTD in both FITC-casein degradation and ATPase assays to determine whether compounds are sequestered by free NTDs, thereby preventing compound-mediated ClpC-WT activation (Figures 3C and 3D). NTD excess reduced the stimulatory effects of all compounds on FITC-casein degradation activities of ClpC-WT/ClpP to varying degrees (Figure 3C). Furthermore, the increase of ClpC ATPase activities by compounds was completely abolished (Figure 3D). We infer that all eight tested compounds deregulate ClpC by binding to its NTD.

**Figure 3:**
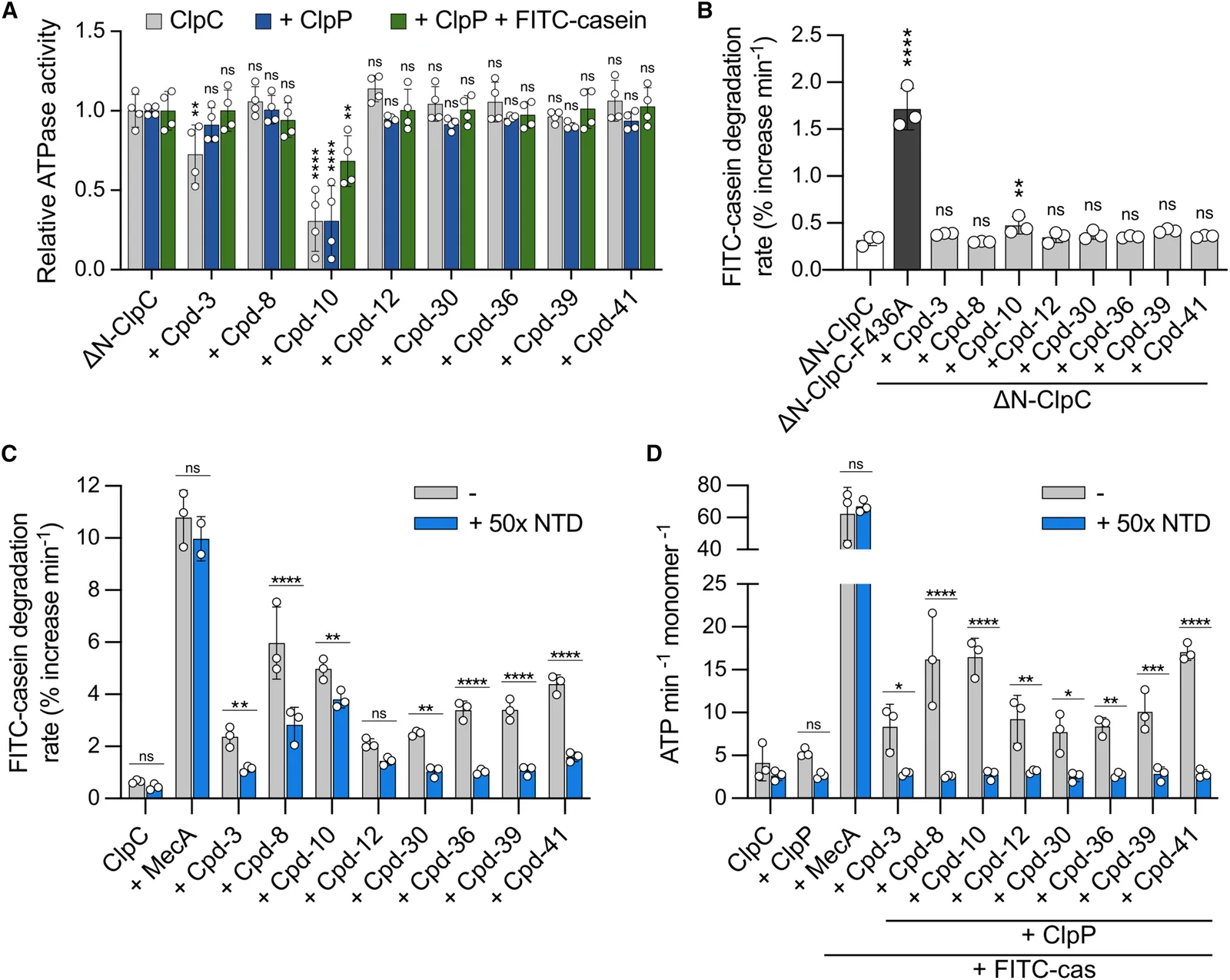
The N-terminal domain (NTD) serves as compound binding site (A) ATPase activities of ΔN-ClpC alone or in the presence of ClpP or ClpP + FITC-casein were determined ± respective compounds (50 μM). Activities were normalized to ΔN-ClpC alone (set to 1) for each condition, *n* = 4. (B) FITC-casein degradation activities of ΔN-ClpC were determined in the presence of ClpP ± respective compounds (50 μM). ΔN-ClpC-F436A was used as a positive control because ΔN-ClpC cannot bind MecA, *n* = 3. (C and D) FITC-casein degradation (C) or ATPase activities (D) of ClpC-WT + ClpP + FITC-casein were determined in absence or presence of a 50-fold excess of isolated NTD at the indicated conditions. Compound concentrations = 25 μM, *n* = 3. All data are represented as mean ± SD. Statistical significance was determined by two-way ANOVA with Dunnett’s post-hoc test (A), one-way ANOVA with Dunnett’s post-hoc test (B), or two-way ANOVA with Holm-Šidák’s post-hoc test (C and D). Positive controls were assessed separately. ns, not significant; ∗*p* < 0.05, ∗∗*p* < 0.01, ∗∗∗*p* < 0.001, ∗∗∗∗*p* < 0.0001.

### Compounds putatively bind to the hydrophobic groove and the pArg1-site of the NTD

To map the compound binding sites on the NTD, we turned to *in silico* docking simulations. First, we used DiffDock-L,^50^^,^^51^ a generative model for ligand docking, to screen for putative ligand binding sites (Figures 4A, 4B, and S6). Strikingly, all compounds were predicted to bind to one of the three known ligand and substrate-binding sites of the NTD, namely the hydrophobic groove and both pArg1/pArg2 binding sites, which independently bind phosphorylated arginines (pArg) as a degron.^14^ Most compounds primarily docked to the pArg1-site, while the hydrophobic groove of the NTD, which is the binding site for all ClpC1-targeting cyclic peptides,^37^^,^^38^^,^^39^^,^^40^ was typically predicted with lower frequencies (Figure 4B). Building on these findings, we performed HADDOCK simulations^52^^,^^53^ biased toward the hydrophobic groove and the pArg1-and pArg2-sites (Figures 4C and S7). In contrast to DiffDock, HADDOCK relies on a well-established, physics-based energy function and a staged workflow (rigid-body docking, semi-flexible refinement, and final refinement in explicit water), which allows local side-chain/backbone adjustments to produce a more complementary ligand-bound conformation. The resulting HADDOCK score is computed as a weighted linear combination of intermolecular energies (e.g., van der Waals, electrostatics, and desolvation) and is commonly used as a physically interpretable proxy for the relative plausibility of a given pose. Moreover, it was designed to be compatible with ambiguous interaction restraints. These *in silico* predictions generally support binding of most compounds to the hydrophobic groove (Cpd-3, Cpd-8, Cpd-12, Cpd-30, Cpd-39) as they yield the lowest HADDOCK score, whereas binding to the pArg2-site is disfavored for all compounds (Figure 4C). However, some predictions showed similar (Cpd-10, Cpd-36) or even better (Cpd-41) average scores for the pArg1-site, qualifying it as an alternative compound binding site.

**Figure 4:**
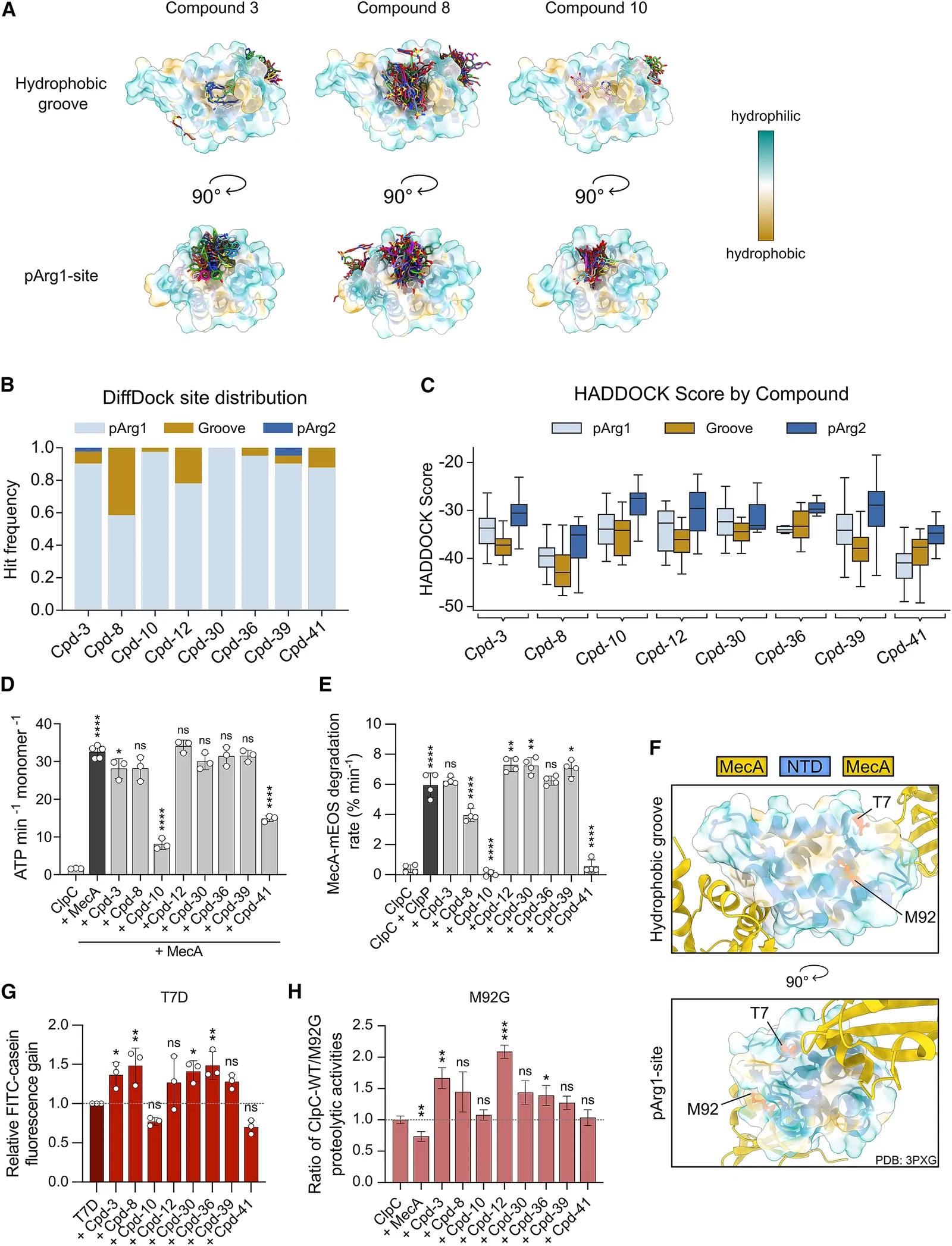
Compounds putatively bind to the hydrophobic groove and pArg1-site of the NTD (A and B) DiffDock docking simulations (*n* = 40) of selected compounds to the ClpC NTD. The NTD is shown as a surface colored by hydrophobicity, two orthogonal views highlighting the hydrophobic groove and the pArg1-site, with all predicted poses displayed for selected compounds (A). Predicted binding poses were clustered by binding site and quantified as the fraction of solutions occupying each site, displayed as stacked bar diagram. (C) Targeted HADDOCK simulations to the hydrophobic groove and both pArg1-/pArg2-sites were performed. Total HADDOCK scores for the corresponding compound docked to each indicated binding site are shown. The solid line in the boxes indicates the median, and the box outlines the 25th and 75th percentiles, respectively. (D) ATPase activities of ClpC were determined in the presence of MecA ± respective compounds (50 μM), *n* = 3. (E) MecA-mEOS degradation by ClpC/ClpP was determined ± respective compounds (50 μM), *n* = 4. (F) Structural representation of ClpC in complex with MecA,^11^ highlighting hydrophobicity of the ClpC-NTD and residues subjected to mutagenesis located at the hydrophobic groove (M92) or the pArg1-site (T7) in red. (G) FITC-casein degradation activities of ClpC-T7D were determined ± respective compounds (50 μM) in the presence of ClpP, *n* = 3. (H) FITC-casein degradation activities of ClpC-WT and ClpC-M92G were determined ± respective compounds (50 μM) in presence of ClpP and normalized to the respective controls. Data represent the factor of the reduced activity by the respective compounds upon M92G mutation (Ratio WT/M92G), *n* ≥ 3. SDs have been propagated from the SDs of degradation activities of WT and M92G under the displayed conditions, respectively. Data in (D)–(E), (G)–(H) are represented as mean ± SD. Statistical significance was determined by one-way ANOVA with Dunnett’s post-hoc test (D and E) (ns, not significant; ∗*p* < 0.05, ∗∗*p* < 0.01, ∗∗∗*p* < 0.001, ∗∗∗∗*p* < 0.0001), with Fisher’s LSD (G) (ns, not significant, *p* = 0.1662 for Cpd-10, *p* = 0.1494 for Cpd-12, *p* = 0.0955 for Cpd-39, *p* = 0.0803 for Cpd-41; ∗*p* = 0.0394 for Cpd-3, ∗*p* = 0.0173 for Cpd-30; ∗∗*p* = 0.0061 for Cpd-8, ∗∗*p* = 0.0079 for Cpd-36), or multiple *t* tests (H) (ns, not significant, *p* = 0.0784 for Cpd-8, *p* = 0.3865 for Cpd-10, *p* = 0.0671 for Cpd-30, *p* = 0.1268 for Cpd-39, *p* = 0.6751 for Cpd-41, ∗*p* = 0.0284 for Cpd-36, ∗∗*p* = 0.0069 for MecA, ∗∗*p* = 0.0026 for Cpd-3, ∗∗∗*p* = 0.00013 for Cpd-12). Positive controls were assessed separately. See also Figures S6–S8.

On this basis, we assessed whether the compounds Cpd-10, Cpd-36, and Cpd-41 could outcompete MecA for binding to the pArg1-site of the NTD. We first determined ClpC ATPase activities in the presence of both MecA and compounds (Figure 4D). The MecA-mediated activation of ClpC ATPase activity was strongly reduced in the presence of Cpd-10 and Cpd-41. As both compounds also enhance ATP hydrolysis, yet to a minor degree as compared to MecA (Figure 1E), this suggests that they prevent MecA association with ClpC. We probed for MecA association with ClpC by using the adaptor variant MecA-mEOS. MecA-mEOS is composed of the C-terminal domain of MecA, which binds to both ClpC pArg1-and pArg2-sites, and the mEOS fluorophore. MecA-mEOS activates ClpC and is subjected to ClpC/ClpP-mediated degradation.^7^ We reasoned that compound binding to the pArg1-site (or pArg2-site) should block MecA-mEOS binding and prevent its degradation. Indeed, Cpd-10 and Cpd-41 effectively abolished MecA-mEOS degradation, indicating they bind to at least one of the pArg-binding sites (Figure 4E). Cpd-8 slightly reduced MecA-mEOS degradation, whereas all other compounds showed no significant inhibition but rather a slight stimulatory effect. To probe for compound binding to the pArg1-site, we used the ClpC mutant T7D, which is located at the pArg1 center (Figure 4F). ClpC-T7D is deregulated and exhibits high autonomous proteolytic activity (Figure S8A).^9^ Intriguingly, most compounds still enhanced ClpC-T7D activity, whereas Cpd-10 and Cpd-41 no longer did so (Figure 4G). These experimental findings support HADDOCK simulations of Cpd-10 and Cpd-41, indicating that these compounds activate ClpC by targeting the pArg1-site.

To clarify the binding sites of the other six compounds, we aimed to mutate the NTD’s hydrophobic groove. Several studies have reported that mutations in the hydrophobic groove of the homologous NTD of Hsp104 strongly decrease domain stability and are thus unsuitable for analysis.^49^^,^^54^^,^^55^ Therefore, we followed a successful approach previously reported for the NTD of Hsp104 and designed the double-mutant ClpC-L14D-I88R to create a salt bridge, which blocks access to the groove^56^ (Figure S8B). However, this mutant could no longer interact with MecA, as evidenced by ATPase and MecA-mEOS degradation assays (Figures S8C and S8D). The mutated sites are not involved in MecA binding, pointing to structural defects. We therefore additionally introduced these mutations into the isolated NTD and determined its melting temperature using nano differential scanning fluorimetry (nanoDSF). The melting temperature massively dropped from 63°C (WT) to 38°C (L14D-I88R) upon NTD mutation (Figure S8E), confirming impaired structural integrity. Hence, we chose Met92 for mutagenesis, which is located at the edge of the hydrophobic groove and has its side chain pointing toward the groove’s center (Figure 4F). FITC-casein degradation and ATPase activities of ClpC-WT and ClpC-M92G in the presence of MecA were similar, indicating that the M92G mutation does not affect the structural integrity of the NTD, which still binds MecA (Figures S8F and S8G). Accordingly, we observed highly efficient degradation of MecA-mEOS by ClpC-M92G/ClpP (Figure S8H). Stimulation of ClpC-M92G proteolytic activity by Cpd-10 and Cpd-41 was comparable to ClpC-WT (Figure 4H), in accordance with previous results suggesting that they bind to the pArg1-site. Enhancement of ClpC-M92G activity by other compounds (Cpd-3, Cpd-12, Cpd-36) was significantly reduced as compared to ClpC-WT (1.27- to 2.09-fold) (Figure 4H), supporting binding of these compounds to or near the hydrophobic groove in agreement with HADDOCK simulations (Figure 4C). The stimulatory factors of other compounds (Cpd-8, Cpd-30, and Cpd-39) were also reduced, yet not statistically significant. We conclude that compounds target two distinct sites in the ClpC NTD: the hydrophobic groove and the pArg1-site.

### Dissection of Cpd-8 binding mode and its potential mechanism of action

To directly determine how the most potent compounds Cpd-8, Cpd-10, and Cpd-41 bind to the ClpC-NTD, we conducted co-crystallization experiments. We could obtain crystal structures of ClpC-NTD with bound Cpd-8 at 1.90 Å and in the apo state at 1.78 Å resolution (Table S1). Interestingly, both datasets have the same space group P4_3_2_1_2 with very similar cell dimensions and one protein molecule per asymmetric unit. Cpd-8 binds to a part of the hydrophobic groove, and the entire ligand shows clear electron density (Figures 5A, 5B, and S9A). A closer look at the binding site reveals that only a part of Cpd-8, dubbed as Cpd-8a (1-(3,5-bis(4-methoxyphenyl)-4,5-dihydro-1H-pyrazol-1-yl)ethan-1-one), forms protein-ligand contacts (Figures S9B–S9D). The other part of the ligand, referred to as Cpd-8b (4-(4-oxo-4,5-dihydro-1H-pyrazolo[3,4-d]pyrimidin-1-yl)benzenesulfonamide), is involved in a crystal contact with a symmetry-equivalent ligand molecule. Notably, protein and ligand form only one hydrogen bond, namely between Gly4 and the carbonyl group of Cpd-8a, whereas all other protein-ligand interactions involve hydrophobic contacts (Figures S9B and S9C). The superposition of the apo and complex structure (Figure S9E) shows a rearrangement of the first three N-terminal residues upon ligand binding. These three residues cover one of the two Cpd-8a methoxyphenyl moieties. Significantly smaller movements can be observed for the Met92 Cε and Tyr80 sidechain. Ligand binding does not induce any other large movements in ClpC-NTD, which is indicated by a low rmsd(C_α_) of 0.14 Å for the superposition of residues 4 to 144 of apo and complex structure.

**Figure 5:**
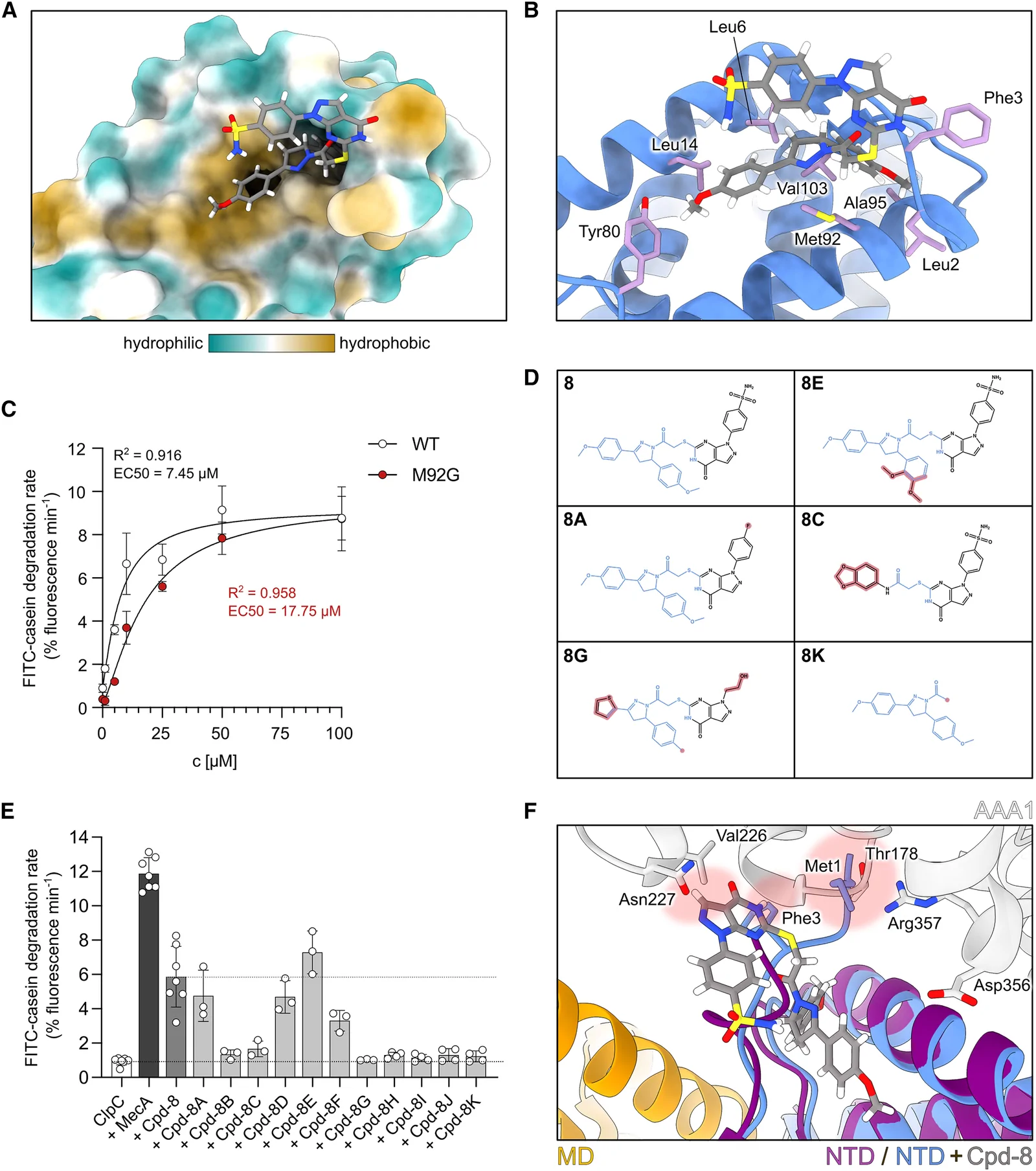
Dissection of Cpd-8 binding mode and its potential mechanism of action (MoA) (A and B) Crystal structure of ClpC-NTD with bound Cpd-8 (carbon atoms in gray) in surface representation colored by hydrophobicity (A) and as cartoon representation (blue) showing selected residues (plum) involved in protein-ligand contacts (B). (C) Dose-response curves of ClpC-WT and ClpC-M92G were determined by monitoring FITC-casein degradation activities at increasing Cpd-8 concentrations in presence of ClpP. EC50 values were obtained by fitting agonist-response data with a four-parameter variable-slope nonlinear regression model. Data are represented as mean ± SD, *n* = 4. (D) Chemical structures of Cpd-8 and selected derivatives. Structural elements of Cpd-8 involved in protein-ligand contacts are displayed in blue. Modifications of respective derivatives are highlighted in red. (E) FITC-casein degradation activities of ClpC ± Cpd-8 and respective derivatives (50 μM) were determined in presence of ClpP. Data are represented as mean ± SD, *n* ≥ 3. (F) The crystal structure of the ClpC NTD (blue) with bound Cpd-8 (carbon atoms in gray) was superimposed on *S. aureus* ClpC in resting state (PDB: 9QCL), AAA1, gray; MD, orange; NTD, purple. Areas with potential steric clashes (VDW overlaps ≥0.6 Å) between the resting state and the N-terminus of ClpC-NTD and bound Cpd-8 are marked in light red. See also Figures S9 and S10 and Table S1.

To biochemically validate the binding position of Cpd-8 observed in the crystal structure, we used the previously described ClpC-M92G mutant, as this residue makes multiple contacts with Cpd-8 (Figures 5B, S9B, and S9C), and determined dose-response curves. The EC50 increased by a factor of 2.4 with the M92G mutation (Figure 5C), indicating decreased affinity in agreement with the determined binding mode.

To assess the functional relevance and evaluate the structure-activity relationship (SAR) of Cpd-8, we tested the efficiencies of commercially available derivatives of Cpd-8 (Figures 5D and S10A) in FITC-casein degradation assays. Modifying one 3-methoxybenzene moiety of Cpd-8a to 1,2-dimethoxybenzene (Cpd-8E) resulted in a slight increase in FITC-casein degradation activity compared to Cpd-8 (Figure 5E). However, more extensive modifications of Cpd-8a generally caused a complete loss of the ability to enhance FITC-casein degradation activities (Cpd-8B, Cpd-8C, Cpd-8G, Cpd-8I). This underlines the central role of Cpd-8a for NTD binding. However, the fragment Cpd-8a alone (Cpd-8K) is insufficient for ClpC activation, indicating that parts of the second moiety, Cpd-8b, also play a crucial role. Substitution of the sulfonamide group with fluoride (Cpd-8A) in the Cpd-8b fragment caused only a slight decrease in proteolytic activity, implying negligible functionality. Replacing Cpd-8b with another large moiety (Cpd-8H) no longer allowed for ClpC activation, indicating that the Cpd-8b fragment cannot simply be swapped out for another bulky group. Thus, we infer that Cpd-8b also contributes to ClpC activation, yet which part of it remains unresolved.

The toxic cyclic peptides cyclomarin A, lassomycin, rufomycin, and ecumicin, which deregulate Mtb ClpC1, bind directly to the center of the hydrophobic groove, making contact with the highly conserved residue Lys85.^37^^,^^38^^,^^39^^,^^40^ This residue forms a salt bridge with Asp356 in the AAA1 domain, which is crucial for resting state formation and activity control, and has been proposed as the reason for ClpC1 deregulation by cyclic peptides.^9^ The binding site of Cpd-8 is slightly different from the previously described cyclic peptides and does not make contact with Lys85 (Figure S10B). We speculate that re-orientation of the ClpC N-terminus could clash with the resting state and destabilize it, leading to ClpC activation. Indeed, superimposing the co-crystal structure with the cryo-EM structure of the ClpC resting state (PDB: 9QCL) revealed serious steric clashes of the N-terminus and the pyrazolo-pyrimidonone moiety of Cpd-8b with the AAA1 domain (Figure 5F), offering a potential mechanistic explanation of how Cpd-8 activates ClpC.

In summary, using co-crystallization, mutagenesis, and compound derivatives, we identified and validated the Cpd-8 binding site, established key structure-activity relationships, and propose a possible mechanism of action.

### Dissection of Cpd-10 binding mode and its potential mechanism of action

HADDOCK simulations and experimental evidence support binding of Cpd-10 to the conserved pArg1-site. This regulatory pocket is particularly interesting because its druggability has not been demonstrated for small molecules so far. Our investigation of Cpd-10 concentrated on the predicted binding conformations from the earlier HADDOCK simulations (Figures 4C and S7), specifically examining the binding poses of the top two docking clusters, 1 and 7. The orientation of the aesculetin moiety (hereafter referred to as Cpd-10a) varied to some degree between the two clusters, while the second half of Cpd-10, 5,6-dimethylthieno[2,3-d]pyrimidine (hereafter referred to as Cpd-10b), interacted with the NTD in a similar manner (Figure 6A). In cluster 1, Cpd-10 forms four hydrogen bonds with the NTD, while in cluster 7, only one hydrogen bond is predicted. In contrast to Cpd-8, both parts of Cpd-10 directly interact with the pArg1-site. Superimposition of the cryo-EM structure of the ClpC resting state with Cpd-10-bound NTD revealed significant steric clashes with the tip of the regulatory MDs (Figure 6B). These clashes involve methyl groups of Cpd-10b with Ala429 and Ala430 in both clusters, and Cpd-10a with Glu426, with the clashes being more pronounced in cluster 1. Glu426 has been linked to the regulation of ClpC activity through stabilizing the resting state via NTD interactions. When mutated (ClpC-E426A), this stabilization is lost, leading to destabilization of the resting state and autonomous ClpC/ClpP proteolytic activity.^8^^,^^9^ We therefore propose that Cpd-10, when bound to the NTD, will clash with the MD tip, triggering ClpC activation.

**Figure 6:**
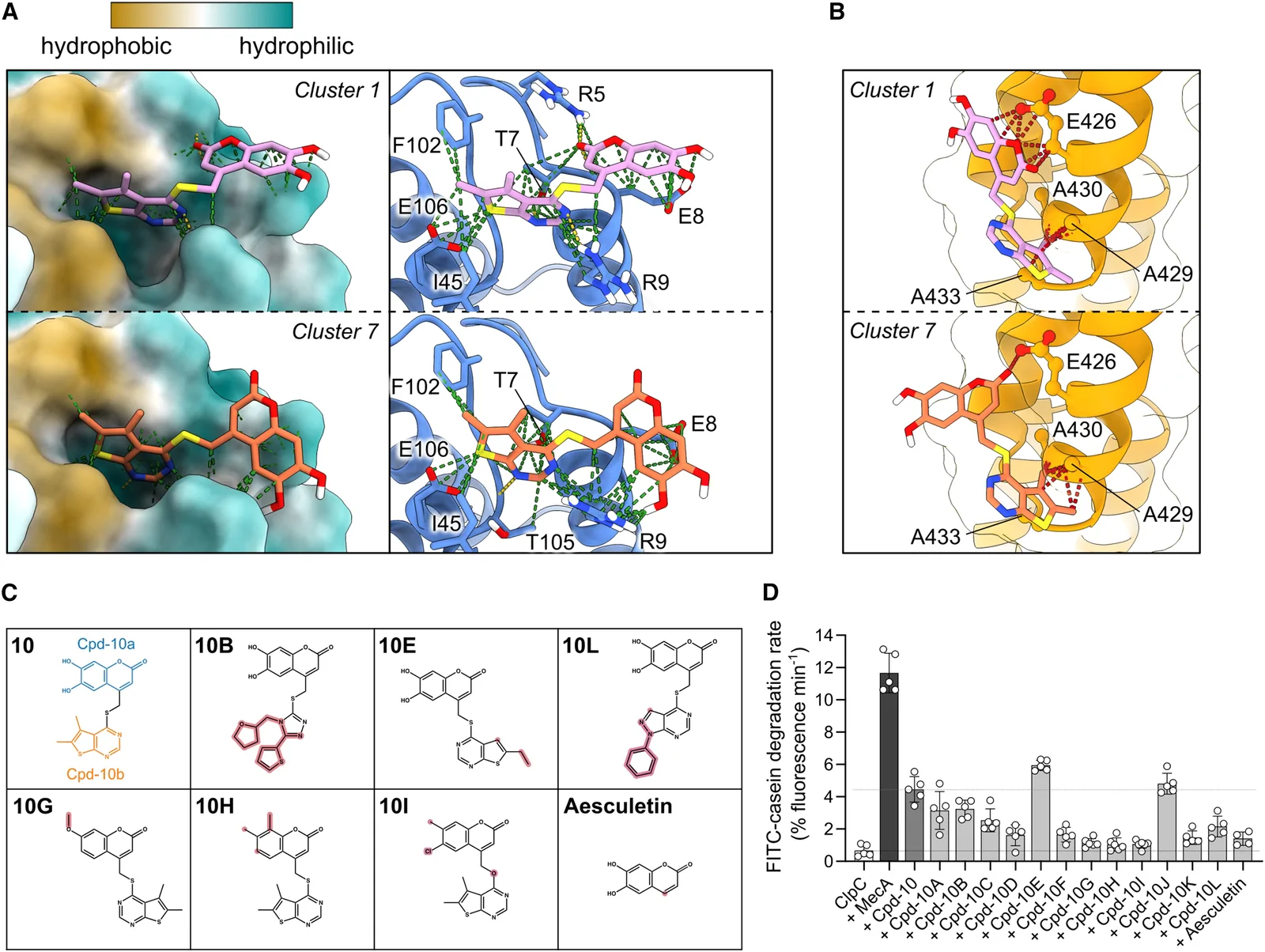
Dissection of Cpd-10 binding mode and its potential mechanism of action (MoA) (A) The top two HADDOCK docking simulations (clusters 1 and 7) of Cpd-10 binding to the NTD are illustrated: a surface representation colored by hydrophobicity (left) and a cartoon view showing hydrogen bonds and contacts (yellow and green dotted lines, respectively, on the right). (B) Superimposition of the cryo-EM structure of the ClpC resting state^9^ with the predicted binding poses of Cpd-10 from clusters 1 and 7. Steric clashes between Cpd-10 and the tip of the regulatory middle domains, including involved residues, are indicated. (C) Chemical structures of Cpd-10 and selected derivatives. Structural elements of Cpd-10 are displayed in blue (Cpd-10a) and orange (Cpd-10b), respectively. Modifications of respective derivatives are highlighted in red. (D) FITC-casein degradation activities of ClpC ± Cpd-10 and respective derivatives (50 μM) were determined in the presence of ClpP. Data are represented as mean ± SD, *n* ≥ 4. See also Figure S11.

To assess whether Cpd-10’s predicted binding poses align with experimental structure-activity relationships, we examined several commercially available derivatives (Figures 6C and S11) using FITC-casein degradation assays and evaluated their activities in the context of the described top two docking clusters 1 and 7. Even slight modifications of Cpd-10a significantly impair or eliminate the derivatives' ability to activate ClpC 3.9- to 9.9-fold, such as Cpd-10G, Cpd-10D, Cpd-10F, Cpd-10H, and Cpd-10I (Figures 6D and S11), highlighting its crucial role in NTD binding. However, Cpd-10a (Aesculetin) alone does not suffice for ClpC activation (Figure 6D), emphasizing the importance of the Cpd-10b moiety for functionality. Although even extensive substitutions of Cpd-10b—such as Cpd-10J, Cpd-10A, Cpd-10C, and Cpd-10B—are generally well tolerated, they cannot be entirely arbitrary. This is evidenced by the decrease in ClpC activity when Cpd-10L and Cpd-10K are present, with reductions of 2.7-fold and 4.1-fold, respectively. On the contrary, removal of the 5-methyl group from the thiophene ring of Cpd-10b and extension of the 6-methyl group to a 6-ethyl group (Cpd-10E) slightly improved compound efficiency (Figure 6D). This shows that Cpd-10b is indeed functionally relevant and needs to satisfy specific criteria.

In light of the SAR results, we assessed the accuracy of HADDOCK clusters 1 and 7, taking into account how respective derivative modifications could affect NTD-binding and affinity, along with potential clashes with the ClpC resting state that could trigger ClpC deregulation. Overall, cluster 1 is strongly favored by the observed effects of Cpd-10 derivatives on FITC-casein degradation activities as the effects of the respective modifications generally correspond well to expected changes in the context of this docking pose. On the contrary, cluster 7 is incompatible with the observed effects for the modifications seen for Cpd-10J, Cpd-10B, and Cpd-10L, as the added or modified (hetero-) aromates would clash extremely with the NTD, yet these derivatives still at least partially activate ClpC/ClpP. Collectively, the SAR trends therefore support cluster 1 as the most consistent binding model.

### Compounds induce ClpC/ClpP-independent toxicity in *S. aureus*

To examine the physiological effects of the compounds as growth inhibitors of *S. aureus*, we monitored growth curves at 37°C using a WT strain (ATCC 25923) and subsequently assessed cell viability through spot dilution series (Figures 7A, 7B, and S12A). All eight tested compounds reduced cell growth and delayed entry into the logarithmic growth phase at high concentrations (200 μM) (Figure 7A). However, their inhibitory impact was minor except for Cpd-10, which fully prevented growth, similar to the well-known antibiotic vancomycin. Cell viability decreased by 1.8–3.7 times across all compounds, except for Cpd-10, which led to a 90-fold reduction (Figures 7B and S12A). We therefore only determined the MIC value of Cpd-10 (64 μg/mL). To document that growth impairment is caused by deregulation of ClpC/ClpP, we tested all compounds on two previously characterized knockout strains (NCTC 8325-4): *clpC::erm*, which has both AAA+ domains deleted through a long in-frame deletion,^17^ and *ΔclpP*.^57^ Unfortunately, the growth of *clpC::erm* and *ΔclpP* was still impaired by compounds, and the inhibitory effects were even worse than observed for the WT strain (Figure 7C). This demonstrates that the toxicity observed across all compounds does not depend on ClpC/ClpP, but rather likely results from effects of unknown origin, which become enhanced in the absence of ClpC/ClpP (Figure 7C). Similar results were obtained at low compound concentrations (10/50 μM), and ClpC-independent growth inhibition was observed for all compounds except Cpd-12 (Figure S12B).

**Figure 7:**
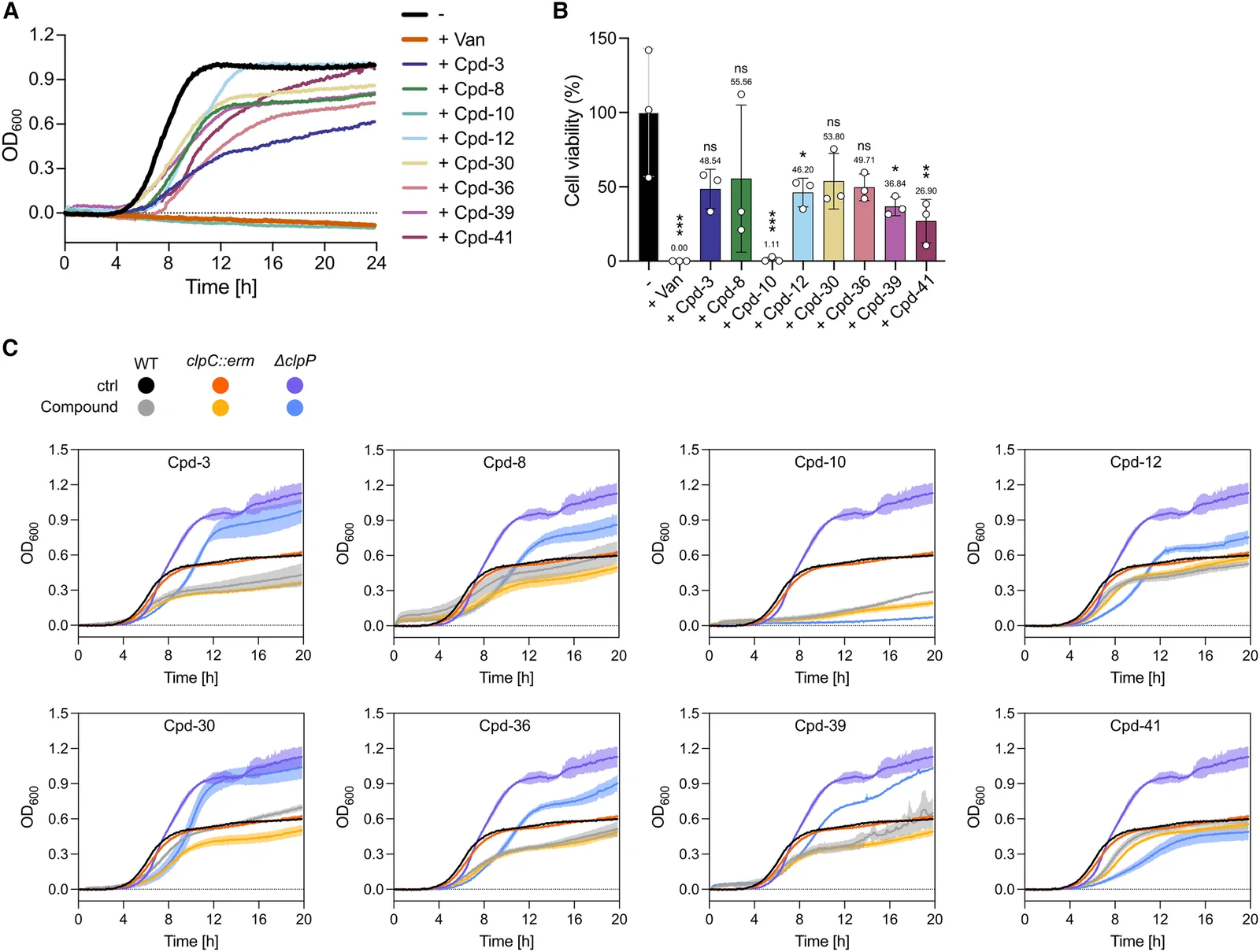
Compounds induce ClpC/ClpP-independent toxicity in *Staphylococcus aureus* (A and B) Growth inhibition of *S. aureus* (WT). Growth curves of *S. aureus* (WT) treated with respective compounds (200 μM) were recorded at 37°C for 24 h (A). Subsequently, cell viability relative to the wild-type was assessed through spot dilution series (B). - indicates DMSO control; Van, Vancomycin; *n* = 3. (C) Growth curves of *S. aureus* (WT) and *S. aureus* knockout strains (*clpC::erm* and *ΔclpP*) treated with respective compounds (200 μM) were recorded at 37°C for 20 h, *n* = 3. In (A), data are represented as mean. In (B)–(C), data are represented as mean ± SD. Statistical significance was determined by one-way ANOVA with Dunnett’s post-hoc test (ns, not significant; ∗*p* < 0.05, ∗∗*p* < 0.01, ∗∗∗*p* < 0.001). See also Figure S12.

Together, our data indicate that although all compounds effectively activate ClpC *in vitro*, they are not yet efficient to cause ClpC-dependent toxicity in *S. aureus* and need further optimization.

## Discussion

In this study, we identify and characterize small molecules that deregulate the ClpC/ClpP protease of *S. aureus*. Using a high-throughput biochemical screen and thorough validation, we demonstrate that *S. aureus* ClpC can be chemically activated by drug-like small molecules. Eight such compounds significantly enhance ClpC-dependent ATP hydrolysis and proteolysis *in vitro*, triggering adaptor-independent and deregulated protein degradation. Such deregulation of an AAA+ protease is expected to create toxicity *in vivo*, allowing for targeting these proteolytic machines even though they are typically not essential for bacterial viability. All eight compounds target the ClpC NTD, underlining its central role in integrating diverse stimuli, including substrates and adaptors, to regulate the AAA+ protein.

Six of the validated compounds likely bind the hydrophobic substrate-binding groove of the ClpC NTD, a site homologous to the cyclic-peptide binding pocket in mycobacterial ClpC1. A co-crystal structure obtained with Cpd-8 confirms engagement of this groove and reveals specific protein-ligand interactions underlying Cpd-8-mediated ClpC activation. The Cpd-8 binding mode deviates from the ones previously described for the cyclic peptides. However, their similar binding site and functional outcome suggest that the NTD hydrophobic groove represents a conserved regulatory hotspot across ClpC homologs, highlighting this site as a broadly exploitable node for chemical intervention in ClpC activity control. The determined Cpd-8 binding site, together with docking analyses, functional mutagenesis, and structure-activity relationship (SAR) studies based on Cpd-8 derivatives, support a model in which hydrophobic-groove ligands mimic aspects of adaptor (MdfA)^10^ or substrate engagement. Cpd-8 putatively destabilizes the inactive ClpC resting state by steric clashes of either the ClpC N-terminal tail with the AAA1 domain or Cpd-8 itself. Further exploration of the co-crystal structure suggests opportunities for structure-guided optimization. In particular, extending Cpd-8a toward the key regulatory residue K85 could enhance both affinity and efficacy.

In addition to the hydrophobic groove, our study identifies the pArg1 pocket as a second, mechanistically distinct site that can be engaged by small molecules to activate ClpC. The pArg1-site recognizes phosphorylated arginines as degrons and functions as allosteric pocket linking degron binding to ClpC activation.^9^^,^^14^ While BacPROTACs exploit this site through pArg-containing ligands,^44^ engagement of the pArg1 pocket by classical small molecules had not been demonstrated. Here, *in silico* docking predictions, targeted mutagenesis, biochemical assays, and structure-activity relationship analysis of Cpd-10 and its derivatives provide converging evidence that this pocket is druggable and that its occupation by small molecules is sufficient to induce ClpC activation. Presumably, Cpd-10 activates ClpC in a manner similar to how arginine-phosphorylated substrates destabilize the resting state, by clashes with the tip of the regulatory middle domain, as previously suggested.^9^ These findings extend the functional repertoire of the pArg1-site and establish it as a viable target for chemical modulation.

While all compounds induce ClpC/ClpP proteolytic activity, they do not lead to the formation of canonical protease complexes. Negative-stain electron microscopy revealed the formation of heterogeneous ClpC/ClpP assemblies upon compound binding. The precise architecture of these complexes varies, however, they are composed of common building blocks including ClpC ring dimers and tetrahedrons among others. Higher-order ClpC/ClpP assemblies were observed irrespective of whether compounds targeted the hydrophobic groove or the pArg1-site. Higher-order assemblies composed of multiple ClpC hexamers have been reported to form in the presence of the toxic cyclic peptide cyclomarin A,^41^^,^^42^ pArg-substrates,^44^ or derepressed ClpC mutants in the presence of the substrate FITC-casein,^9^ respectively. These observations suggest that different regulatory sites all share a common ClpC activation pathway that is closely linked to assembly formation.

An important feature of the small-molecule activators described here is their capacity to sustain ClpC-ClpP proteolytic activity over time. Unlike the adaptor protein MecA, which is rapidly degraded as part of an intrinsic negative feedback loop on ClpC activation, these compounds are not depleted in the process. Consequently, they enable longer-lasting protease activity and higher overall substrate breakdown. This highlights a fundamental difference between natural adaptor regulation and chemical deregulation, with promising implications for the controlled manipulation of bacterial proteostasis. Notably, *M. tuberculosis* encodes for ClpC2 and ClpC3, which harbor domains homologous to the ClpC1 NTD. ClpC2 and ClpC3 can sequester cyclic peptides,^45^^,^^58^ thereby protecting ClpC1 from drug-induced deregulation. Such a mechanism is not expected to exist for compounds targeting *S. aureus* ClpC.

Despite robust biochemical activation of ClpC, the compounds exhibited antibacterial activity against *S. aureus* that was independent of ClpC or ClpP in cellular assays. While this precludes a direct link between ClpC activation and growth inhibition in *S. aureus*, it does not detract from the mechanistic insights provided by this study. Rather, it highlights the challenges inherent in translating *in vitro* protease modulation into cellular phenotypes and underscores the need to further optimize selectivity and cellular engagement. In the case of the most toxic Cpd-10, the observed ClpC-independent toxicity may reflect off-target effects mediated by its aesculetin moiety, which is a coumarin derivative. Interestingly, aminocoumarins are natural antibiotics that inhibit DNA gyrase.^59^ Although these potential off-target interactions were not directly assessed here, they provide a plausible explanation for the observed cellular growth inhibition. Given the dual activating and inhibiting nature of Cpd-10 on ClpC activity and the fact that inhibition of ClpC1 is toxic in mycobacteria, it would be interesting to evaluate Cpd-10 and related derivatives in mycobacterial systems, where engagement of the essential ClpC1 homolog may yield distinct cellular outcomes.

More broadly, our findings contribute to a growing perception of AAA+ proteases as chemically addressable molecular machines whose activity can be tuned through allosteric regulation. By mapping two distinct ligandable sites within the ClpC NTD and linking their engagement to a defined functional outcome, this work advances the understanding of ClpC activity control mechanisms and expands the toolkit for chemical biology approaches to target this bacterial PQC component. These insights may inform future efforts to design chemical compounds or degradation strategies targeting ClpC.

### Limitations of the study

Direct binding of most compounds to the ClpC NTD remains to be determined. While *in silico* predictions and diverse independent experimental evidence indicate the compounds bind to the NTD, direct evidence still needs to be provided. This should also be used to determine dissociation constants, adding important information to the so far determined EC50 values. While the small molecules identified in this study effectively activate *S. aureus* ClpC *in vitro*, their *in vivo* effectiveness appears to be ClpC/ClpP-independent. This discrepancy may stem from reduced uptake due to membrane impermeability or off-target effects. Additionally, their relatively low to moderate affinity may not be sufficient to significantly disrupt proteostasis by deregulating ClpC *in vivo*. Clarifying the first issue could involve measuring intracellular compound concentrations, and off-targets could be identified using techniques like photo-crosslinking probes. Furthermore, enhancing binding affinities to ClpC, guided by structural insights into SARs and the proposed MoA for Cpd-8 and Cpd-10, should be a priority to optimize compounds and achieve ClpC-dependent toxicity while minimizing off-target effects. In support of such attempts, further structural information about the respective compound binding modes is required, as most of the evidence provided here is indirect and mostly based on *in silico* prediction models.

Moreover, our study did not investigate whether these compounds reduce biofilm formation and virulence in a host-infection model, which would provide valuable insights into their therapeutic potential. Thus, future studies should aim to assess the efficacy of optimized ClpC activators in relevant infection models to determine their potential as antivirulence agents and to better understand the role of ClpC in *S. aureus* pathogenesis.

Regarding Cpd-10, its binding to an unknown second site that might counteract ClpC activation warrants caution and should be considered in future drug development. Elucidation of the nature of this secondary binding site and its potential impact on ClpC activity will be essential for the rational design of improved compounds.

## Resource availability

### Lead contact

Requests for further information and resources should be directed to and will be fulfilled by the lead contact, Axel Mogk (a.mogk@zmbh.uni-heidelberg.de).

### Materials availability

This study did not generate new unique reagents or microbial strains.

### Data and code availability

• Coordinates and structure factors of the two X-ray structures reported in this study are deposited at the Protein DataBank PDB under accession codes 9TXC (ClpC-NTD apo) and 9TXD (ClpC-NTD:Cpd-8). Coordinates of ClpC-MecA (3PXG), ClpC resting state (9QCL), ClpC1-NTD-CyclomarinA (3WDC), ClpC1-NTD-Lassomycin (8IBP), ClpC1-NTD-Rufomyin (6CN8), and ClpC1-NTD-Lassomycin (8IBP) structures are deposited at the Protein DataBank PDB under the indicated accession codes.
• This article does not report original code.
• Any additional information required to reanalyze the data reported in this study is available from the lead contact upon request.

## Acknowledgments

T.J. was supported by the 10.13039/501100021374Heidelberg Biosciences International Graduate School (HBIGS). This work was supported by a grant of the 10.13039/501100001659Deutsche Forschungsgemeinschaft to A.M. (MO970/8-1). We acknowledge the support by the grant “Compound Screening” (ExU 6.1.5) by the Flagship Initiative “Engineering Molecular Biosystems” of Heidelberg University. I.S., M.S., and J.K. acknowledge the service SDS@hd, supported by the Ministry of Science, Research and Arts Baden-Württemberg, and the 10.13039/501100001659DFG through grant INST 35/1503-1 FUGG. We acknowledge access to ESRF Grenoble beamline ID30A-1 (MASSIF-1) and thank the ESRF staff for their support. We thank Britta Klem from the CCTP/BZH crystallization platform for excellent technical support. We are grateful for access to the infrastructure and support provided by the Cryo-EM Network at Heidelberg University (HDcryoNet). For the publication fee we acknowledge financial support by Heidelberg University. We thank Dorte Frees for providing the *S. aureus* knockout strains used in this study. Lastly, we thank Alexander Dalpke for generously providing laboratory space and infrastructure to conduct S2 experiments.

## Author contributions

Conceptualization, T.J., B.B., and A.M.; methodology and investigation, T.J., V.V., B.J., M.S., J.K., D.F., and A.M.; formal analysis, T.J., V.V., U.U., M.S., J.K., D.F., and A.M.; resources, U.U., J.K., D.F., E.S., I.S., B.B., and A.M.; writing – original draft, T.J.; writing – review and editing, T.J., V.V., U.U., J.K., D.F., E.S., and A.M.; supervision, A.M.; visualization, T.J., V.V., J.K., and A.M.; funding acquisition, B.B. and A.M.

## Declaration of interests

The authors declare no competing interests.

## STAR★Methods

### Key resources table

**Table:** 

REAGENT or RESOURCE	SOURCE	IDENTIFIER
**Bacterial and virus strains**		

*E. coli* XL1 blue	Agilent	200249
*E. coli* BL21 Rosetta DE3	Novagen	70954–3
*E. coli ΔclpB* MC4100 Kanamycin sensitive	Jenne et al.^9^	N/A
*Staphylococcus aureus*	ATCC	ATCC 25923
*Staphylococcus aureus clpC::erm*	Frees et al.^17^	NCTC 8325–4
*Staphylococcus aureus ΔclpP*	Frees et al.^57^	NCTC 8325–4

**Chemicals, peptides, and recombinant proteins**		

For a list of used compounds, see Table S4		N/A
Kanamycin	Sigma	K4000
Ampicillin	Roth	K029.2
Protino™ Ni-IDA	Macherey-Nagel	MN745210.30
Protease inhibitor Pepstatin A	Roth	2936.3
Protease inhibitor Aprotinin	Roth	A162.3
Protease inhibitor Leupeptin	Roth	CN33.4
DNaseI	Roche	10104159001
Phusion HF DNA-Polymerase	ThermoScientific	F-530
T4 DNA Ligase	NEB	M0202L
Imidazole	Roth	3899.4
Pyruvate kinase	Sigma	P7768
FITC-casein (Casein fluorescein isothiocyanate)	Sigma	C0528
Bradford reagent	BioRad	5000006
ATP	Sigma	A26209
NADH	Sigma	43420
PK/LDH	Sigma-Aldrich	P0294
PEP (Phospho(enol)pyruvic acid trisodium salt hydrate)	Sigma	P7002
ATPγS	Roche	11162306001
Uranyl acetate	Ted Pella	19481
IPTG (Isopropyl-β-D-1-thiogalactopyranoside)	Roth	CN08.3
DMSO	Sigma	276855
ADEP1	Heike Brötz-Oesterhelt	N/A
SYPRO Ruby Protein Gel Stain	Thermo Fisher	S12000
Vancomycin	Sigma	V2002

**Deposited data**		

ClpC-NTD apo structure	This paper	PDB:9TXC
ClpC-NTD:Cpd-8 structure	This paper	PDB:9TXD
ClpC-MecA structure	Wang et al.^11^	PDB:3PXG
ClpC resting state structure	Jenne et al.^9^	PDB:9QCL
ClpC1-NTD + Cyclomarin A structure	Vasudevan et al.^38^	PDB:3WDC
ClpC1-NTD + Lassomycin structure	Jagdev et al.^37^	PDB:8IBP
ClpC1-NTD + Rufomycin structure	Wolf et al.^40^	PDB:6CN8
ClpC1-NTD + Ecumicin structure	Wolf et al.^39^	PDB:6PBS

**Oligonucleotides**		

For primers used for cloning, mutagenesis and sequencing, see Table S3	This paper	N/A

**Recombinant DNA**		

For used plasmids, see Table S2	This paper	N/A

**Software and algorithms**		

FIJI	Schindelin et al.^60^	https://imagej.net/software/fiji/downloads
Prism 10	Graphpad Software	https://www.graphpad.com/updates
ChimeraX 1.10	UCSF, Goddard et al.,^61^ Pettersen et al.^62^	https://www.cgl.ucsf.edu/chimerax/download.html
Prometheus Panta Analysis software (version 1.4.3)	NanoTemper	N/A
EPU software	ThermoFisher	N/A
EMAN2 v.2.12	Tang et al.^63^	https://cryoem.bcm.edu/downloads/view_eman2_versions
IMAGIC-4D	van Heel et al.^64^	https://download.imagescience.de/download.html
XDS	Kabsch.^65^	N/A
AIMLESS as part of the CCP4 software package	Evans et al.,^66^ Winn et al.^67^	N/A
PHASER implemented in the Phenix package	McCoy et al.,^68^ Liebschner et al.^69^	N/A
AlphaFold2	Jumper et al.^70^	N/A
phenix.elbow	Moriarty et al.^71^	N/A
Coot	Emsley et al.^72^	N/A
phenix.refine	Afonine et al.^73^	N/A
MolProbity	Chen et al.^74^	N/A
Ligplot+ v2.3	Laskowski et al.^75^	N/A
DiffDock-L	Corso et al.^50^^,^^51^	N/A
HADDOCK 2.4	Honorato et al.^52^^,^^53^	N/A
ChemDraw v.22.2.0	Revvity Signals	N/A

**Other**		

French Pressure cell press	Sim-Aminco	N/A
Superdex S200	Cytiva	28–9893–35
CLARIOstar^plus^ plate reader	BMG Labtech	N/A
Prometheus^TM^ Panta	NanoTemper	N/A
Prometheus^TM^ standard capillaries	NanoTemper	PR-C002
ÄKTA pure	Cytiva	N/A
Superose 6 Increase 10/300 GL column	Cytiva	29–0915–96
Talos L120C electron microscope	Thermo Fisher Scientific	N/A
GloQube	Quorum	N/A
MultiDrop device	ThermoFisher Scientific	N/A
FlexDrop with plate stacker	Revvity	N/A
Envision HTS reader	Revvity	N/A
Victor Nivo microplate reader	Perkin Elmer	N/A

### Experimental model and study participant details

*Escherichia coli* and *Staphylococcus aureus* strains used in this study are listed in Table S2 and have been described before.^7^^,^^8^^,^^9^^,^^41^

### Method details

#### Protein purification

*Staphylococcus aureus* (*Sa*) ClpC (WT and derivatives), ClpP, and MecA were purified from *E. coli ΔclpB::kan* cells overexpressing them using pDS56-derived expression vectors.^8^ *Escherichia coli* (*E. coli*) cells were cultivated in LB medium at 30°C with agitation at 120 rpm, supplemented with appropriate antibiotics. For protein overexpression, 2x YT medium (16 g/L tryptone, 10 g/L yeast extract, 5 g/L NaCl, adjusted to pH 7) was used. *E. coli* XL1 Blue was utilized for cloning and retaining of plasmids requiring kanamycin at 50 μg/mL and ampicillin at 100 μg/mL for plasmid propagation.

ClpC deletion mutants were generated by PCR, and point mutants were constructed by Quikchange one-step site-directed mutagenesis. All mutations were verified by sequencing. ClpC, ClpP, MecA, FtsZ, MecA-mEOS3.2, and isolated ClpC-NTD harbor a C-terminal His6-tag and were purified using Ni-IDA (Macherey-Nagel) following the instructions provided by the manufacturer. In short, cell pellets were resuspended in buffer A (50 mM NaH_2_PO_4_, 300 mM NaCl, 5 mM β-mercaptoethanol, pH 8.0) supplemented with protease inhibitors (Roche). After cell lysis using a French press, the cell debris was removed by centrifugation at 18,000 g for 60 min at 4°C, and the cleared lysate was subsequently transferred into a plastic column containing pre-equilibrated 0.8–1 g Protino Ni-IDA resin (Macherey-Nagel) at 4°C. Afterward, the resin was washed once with buffer A. His-tagged proteins were eluted by adding buffer A supplemented with 250 mM Imidazole. Subsequently, pooled protein fractions were subjected to size exclusion chromatography (Superdex S200, Amersham) in buffer B (50 mM Tris-HCl, pH 7.5, 50 mM KCl, 20 mM MgCl_2_, 5% (v/v) glycerol, 2 mM DTT).

Pyruvate kinase of rabbit muscle and Casein fluorescein isothiocyanate (FITC-casein) from bovine milk were purchased from Sigma. Protein concentrations were determined with the Bradford assay (BioRad).

#### High-throughput screen (HTS)

The HTS was performed using the Diversity Library maintained by the Chemical Biology Core Facility at the European Molecular Biology Laboratory (EMBL; (https://www.embl.org/groups/chemical-biology/screening/). 337 assay plates (384-well format) were screened in 7 batches. These plates are pre-plated with compounds in columns 3 to 22 (1 μL of 400 μM compound solution in 4% DMSO) and 1 μL of 4% DMSO solution in columns 1 and 2. In columns 1 to 22, 4 μL of ClpP/ClpC reaction mix was added using a MultiDrop device (ThermoFisher Scientific); in columns 23 and 24, the 4 μL of reaction mix contained MecA as well and served as the positive control. The reaction was started by adding 5 μL of an ATP solution (Buffer: 50 mM Tris pH 7.5, 20 mM KCl, 10 mM MgCl_2_, and 2 mM DTT; ATP, Phosphoenolpyruvate (PEP), and pyruvate kinase (PK)) with a FlexDrop (Revvity, with plate stacker) to a final assay volume of 10 μL. The final ingredient concentrations in each well were as follows: 0.4% DMSO, 0.5 μM ClpC, 0.75 μM ClpP, 0.5 μM FITC-casein, 2 mM ATP, 3 mM PEP, 200 ng PK, and 0.75 μM MecA in the positive control wells. Compounds were screened at a single concentration (40 μM). After 60 min of incubation, the fluorescence signal (Excitation 485 nm/Emission 535 nm) was read with the Envision HTS reader from Revvity (with plate stacker). For each batch, 2 QC-plates (plates with no screening compounds in column 3–22) were screened.

#### Biochemical assays

Buffer C (50 mM Tris-HCl, pH 7.5, 50 mM KCl, 20 mM MgCl_2_, 2 mM DTT) was used for all biochemical assays. Compounds were added to a final concentration of 50 μM with 1% (v/v) DMSO unless stated otherwise.

#### ATPase assay

Rates of ATP hydrolysis were determined using a coupled-colorimetric assay as described previously.^76^ Oxidation of NADH to NAD^+^ was monitored by measuring absorbance at 340 nm.

ATPase activity measurements were performed at final concentrations of 0.5 μM ClpC, 0.75 μM ClpP, 0.75 μM MecA, 0.5 μM FITC-cas, and an ATP regenerating system (PK/LDH mix: 250 μM NADH, 500 μM PEP, 1/20 (v/v) PK/LDH (Sigma-Aldrich)) in buffer C supplemented with 2 mM ATP at 30°C in a transparent 96-well plate (Greiner) using a CLARIOstar^plus^ plate reader (BMG Labtech). Isolated ClpC-NTD was added to a final concentration of 25 μM.

The ATP hydrolysis rate for at least three biological replicates was calculated according to the following equation:

$$\text{ATPase}\;\text{rate}=\frac{1}{\varepsilon_{\text{NADH}}\times C_{\text{ClpC}}\times d}\times \frac{\Delta A_{340\;\text{nm}}}{\Delta t}$$

ε_NADH_ – molar absorption coefficient of NADH at a wavelength of 340 nm (6220 M^−1^cm^−1^).

C_ClpC_ – final concentration of ClpC (0.5 μM).

d – optical path length (1 cm).

ΔA_340 nm_/Δt – slope of the linear decline in absorption at a wavelength of 340 nm.

#### Degradation assays

Proteolytic activity was determined using fluorescein-labelled casein (FITC-casein) and FtsZ at final concentrations of 0.5 μM ClpC, 0.75 μM ClpP, 0.75 μM MecA, 0.5 μM FITC-casein, 1 μM FtsZ, 0.5 μM MecA-mEOS3.2, and an ATP regenerating system (3 mM PEP, 20 ng/μL PK (Sigma-Aldrich)) in buffer C. Isolated ClpC-NTD was added to a final concentration of 25 μM. ADEP1 was used in FITC-casein degradation assays at a concentration of 10 μg/mL. Reactions were started by the addition of ATP to a final concentration of 2 mM.

The increase of fluorescence upon FITC-casein degradation was monitored at 483–14 nm and 530–30 nm as excitation and emission wavelengths, respectively, in black 96-well plates (Greiner) using a CLARIOstar^plus^ plate reader (BMG Labtech). The increase of fluorescence upon MecA-mEOS degradation was monitored at 489–14 nm and 529.5–17 nm as excitation and emission wavelengths, respectively, in black 96-well plates (Greiner) using a CLARIOstar^plus^ plate reader (BMG Labtech). The raw fluorescence signal was normalized by setting the initial value to 100%. The FITC-casein fluorescence gain (% min^−1^) was calculated by determining the initial slopes of the linear fluorescence signal increase for at least three biological replicates. Cpd-10 presence decreased total FITC-casein fluorescence.

FtsZ degradation was monitored by SDS-PAGE followed by Coomassie staining. Band intensities were quantified using FIJI.^60^

#### Analytical size-exclusion chromatography (SEC)

Complex formation of ClpC and respective variants was monitored via analytical size-exclusion chromatography. SEC was performed at 4°C using an ÄKTA pure (Cytiva) equipped with a Superose 6 Increase 10/300 GL column (Cytiva), pre-equilibrated with buffer B supplemented with 2 mM ATP. 6 μM ClpC-E280A/E618A (ClpC-DWB) in the presence of 9 μM ClpP and 6 μM FITC-casein was pre-incubated with 2 mM ATPγS and 50 μM of respective compounds at RT for 5 min, and 200 μL of the sample were loaded onto the column. Aliquots were taken and subjected to SDS-PAGE analysis. Gels were stained using SYPRO Ruby Protein Gel Stain. For each sample containing a compound, two biological replicates were performed.

#### Nano differential scanning fluorimetry (nanoDSF)

Domain stability was assessed via nano differential scanning fluorimetry (nanoDSF). 100 μM ClpC-NTD-WT and L14D-I88R in 50 mM HEPES (pH 7.5), 50 mM KCl, and 2 mM DTT were loaded into Prometheus™ high sensitivity capillaries (NanoTemper). Thermal unfolding was analyzed by gradually heating from 20°C to 95°C at a rate of 0.5 °C/min with a Prometheus™ Panta device (NanoTemper), while tracking turbidity and the intrinsic fluorescence at 330/350 nm. The melting temperature (T_m_) was determined using the Prometheus Panta Analysis software (version 1.4.3). Three technical replicates were performed for each sample.

#### Negative-stain electron microscopy (nsEM)

Negative staining, data collection, and processing were performed as described previously.^77^ In brief, a 5 μL sample was applied to a glow-discharged grid with a 6–8 nm thick layer of continuous carbon. After incubation for 5–60 s, the sample was blotted on a Whatman filter paper 50 (1450–070) and quickly washed with three drops of water. Samples on grids were stained with 2.5% aqueous uranyl acetate. Images were acquired using a ThermoFisher Talos L120C electron microscope equipped with a Ceta 16M camera, operated at 120 kV. The micrographs were acquired at ×36,000 (Cpd-8, Cpd-10) or ×57,000 (Cpd-41) magnification (resulting in 4.13/2.55 Å per pixel, respectively) using EPU software. For 2D classification of ClpC-DWB + ClpP + FITC-casein + Cpd-8, 6,925 particles were selected using the boxing tool in EMAN2 (box pixel size 360).^63^ Image processing was carried out using the IMAGIC-4D package.^64^ Particles were band-pass filtered, normalized for gray value distribution, and mass centered. 2D alignment, classification, and iterative refinement of class averages were performed as previously described.^78^ Following classification, particles were sub-classified to further improve the data quality of selected classes.

Samples were prepared for and subjected to analytical SEC as described previously, with the peak fraction of the early eluting large complexes being applied to a grid.

#### Bacterial growth inhibition and viability assays

*S. aureus* strains (WT: ATCC 25923, *clpC::erm*/*ΔclpP*: NCTC 8325–4) were cultured on Columbia Agar plates containing 5% Sheep Blood (BD Biosciences). For assay preparations, single colonies were transferred into trypticase soy bouillon (TSB; BD, Germany) and grown overnight at 37°C with shaking at 150 rpm. The following day, the bacterial cells were diluted to an OD_600_ = 0.05, grown in TSB until reaching exponential growth (OD_600_ = 0.6–0.8), and then diluted to a final concentration of 1 x 10^5^ CFU/mL in a polystyrene U-bottom 96-well plate (Greiner) containing respective compounds (10/50/200 μM, as specified) at a final DMSO concentration of 2% (v/v). The plate was sealed with a gas-permeable Breathe-Easy sealing membrane (Sigma). Wells supplemented with Vancomycin and DMSO were included as control groups, respectively. Growth curves (OD_600_) were recorded at 37°C with shaking at 600 rpm (linear shaking) for 20–24 h using a Victor Nivo microplate reader (PerkinElmer). Subsequently, the bacterial cells were adjusted to OD_600_ = 1, and serial dilutions (10^−1^ - 10^−6^) were spotted on Columbia Agar plates containing 5% Sheep Blood using a custom-made spotter (ZMBH workshop, University of Heidelberg) to assess cell viability. Plates were incubated overnight at 37°C. Three biological replicates were performed.

The MIC value of Cpd-10 was determined according to EUCAST/ISO 20776–1 methodology. *S. aureus* ATCC 25923 was streaked on blood agar and colonies were resuspended in saline (0.85% (w/v) NaCl). The resuspended cells were adjusted to 0.5 McFarland and used at 5 x 10^5^ CFU/ml final concentration in Cation-adjusted Mueller-Hinton Broth (CAMHB) as growth medium. Growth inhibition was determined in the presence of serial dilutions of Cpd-10 (0.5–256 μg/mL). Respective serial dilutions of Vancomycin served as control for growth inhibition. The final DMSO concentration was 1% (v/v). Cells were incubated in 96-well plates at 37°C for 18 h and bacterial growth was checked by visual inspection. The MIC value was determined based on three biological replicates.

#### Crystallization, data collection, model building, and refinement

Crystals were grown at 291.15 K using sitting drop vapor diffusion. The crystallization reservoirs were composed of 2.145 M (NH_4_)_2_SO_4_, 0.1 M MES pH 6 (ClpC-NTD apo) and 1.909 M (NH_4_)_2_SO_4_, 0.1 M Tris pH 8 (ClpC-NTD:Cpd-8). Crystallization drops contained 100 nL reservoir solution and 500 nL protein at a concentration of 7.5 mg/mL. Crystals grew within 14–21 days. Crystals were frozen in liquid nitrogen using glycerol as cryo-protectant. Data were collected at ESRF beamline ID30A-1 at cryogenic conditions, integrated using XDS,^65^ and scaled using AIMLESS^66^ as part of the CCP4 software package.^67^ Phases were obtained by molecular replacement with PHASER^68^ implemented in the Phenix package^69^ using an AlphaFold2^70^ prediction as search model. The resulting datasets had space group P4_3_2_1_2, a maximum resolution of 1.78 Å (ClpC-NTD apo) and 1.9 Å (ClpC-NTD:Cpd-8) with one ClpC-NTD molecule per asymmetric unit each. Cpd-8 was parametrized from SMILES description using phenix.elbow.^71^ Iterative model building and refinement were performed with Coot^72^ and phenix.refine.^73^ The quality of the resulting structural models was analyzed with MolProbity.^74^ Figure S8A was prepared with PyMol 3.1.6.1 (The PyMol Molecular Graphics System, Schrödinger). Figure S8B was prepared using Ligplot+ v2.3.^75^ Crystallographic data are summarized in Table S1. Coordinates and structure factors of the two X-ray structures reported in this study are deposited at the Protein DataBank PDB under accession codes 9TXC (ClpC-NTD apo) and 9TXD (ClpC-NTD:Cpd-8).

#### *In silico* predictions

##### DiffDock-L screening for binding site prediction

In order to locate the binding sites and pre-screen for plausible binding poses, we docked all compounds to the N-terminal domain (NTD) of ClpC (apo-ClpC) using DiffDock-L.^50^^,^^51^ DiffDock is a diffusion-based generative model with a graph neural network architecture trained to predict docking poses given a protein target and a ligand. Although DiffDock can generate poses that violate physical constraints^79^ and is not trained with explicit energy or force-field terms, it remains suited for an initial prescreening stage. Its learned generative and scoring model captures patterns of geometric complementarity between ligands and protein pockets, which can be used for the identification of structurally plausible binding regions.

For each compound, we sampled 40 binding poses with the locally installed DiffDock-L (github commit: 9a22cbc) using 40 diffusion timesteps. Most of DiffDock-L predictions converged to three known binding sites in the ClpC-NTD: pArg1, NTD, and pArg2. We then quantified how often DiffDock-L positioned ligands in each of the three sites. For every sampled pose, we computed the center of mass (CoM) of a ligand and computed its Euclidean (L2) distance to the site-defining residue (E106, I88/M92, E32) sidechain atoms. Each pose was assigned to the site whose sidechain atoms were closest (minimum heavy-atom distance); poses with a minimum distance >10 Å to all side-chain atoms were excluded from further analyses.

##### Biased flexible docking of ligands with HADDOCK 2.4

To obtain physically consistent ligand binding modes, we performed restrained docking with HADDOCK 2.4 using experimental restraints and binding sites obtained with DiffDock-L. For each ligand, we specified a set of active residues (pArg1: T7/E106, Hydrophobic groove: Y80/M92, pArg2: E32) to bias sampling toward the intended binding site. Docking was run in three stages: 1000 structures for rigid-body docking, 200 structures for semi-flexible refinement, and 200 structures for final water refinement. All parameters were kept at their default values, as defined by the developers of HADDOCK, except the electrostatics weight in the HADDOCK score computation, which was set to 0.1. Each ligand was independently docked to all three candidate binding sites, and the median HADDOCK score was computed for the resulting clusters. Final poses shown in the main text were selected based on the total HADDOCK score and visual inspection.

##### Bioinformatic analyses

Structural figures were prepared with ChimeraX v1.10.^61^^,^^62^ Chemical structures were prepared using ChemDraw v22.2.0.

### Quantification and statistical analysis

Quantitative data were analyzed and graphs generated using GraphPad Prism (GraphPad Software), unless otherwise indicated. Microsoft Excel was used to determine slopes from ATPase and degradation assay measurements. Statistical tests, sample sizes (n), and data presentation are detailed in the respective figure legends, while the definitions of technical and biological replicates are provided in the corresponding Method Details. Data are presented as mean ± SD unless otherwise indicated. Statistical significance was defined as *p* < 0.05. Statistical analyses included one- and two-way ANOVA with appropriate post-hoc tests (Dunnett’s or Holm-Šidák’s), unpaired Student’s t-tests, multiple t-tests, Fisher’s LSD, two-tailed Pearson correlation, and four-parameter variable-slope nonlinear regression for EC50 determination, as specified in the figure legends. Because the positive control served as a reference for assay validation, comparisons involving the positive control were performed separately using unpaired Student’s t-tests to reduce the risk of type II error; compound treatments were compared with the negative control.
