# Supplementary material for: Small-molecule activators of the Staphylococcus aureus ClpC/ClpP AAA+ protease
Source: iScience. 2026 Sep 22;29(10):117738. doi: 10.1016/j.isci.2026.117738 (PMC13657355; doi:10.1016/j.isci.2026.117738)
Supplement: Supplementary file 1 [file mmc1.pdf]

iScience, Volume *29*

Supplemental information

Small-molecule activators

# of the *Staphylococcus aureus*

ClpC/ClpP AAA+ protease

Timo Jenne, Vsevolod Viliuga, Ulrike Uhrig, Britta Jehle, Merlin Schwan, Jürgen Kopp, Dirk Flemming, Elisabeth Seebach, Irmgard Sinning, Bernd Bukau, and Axel Mogk

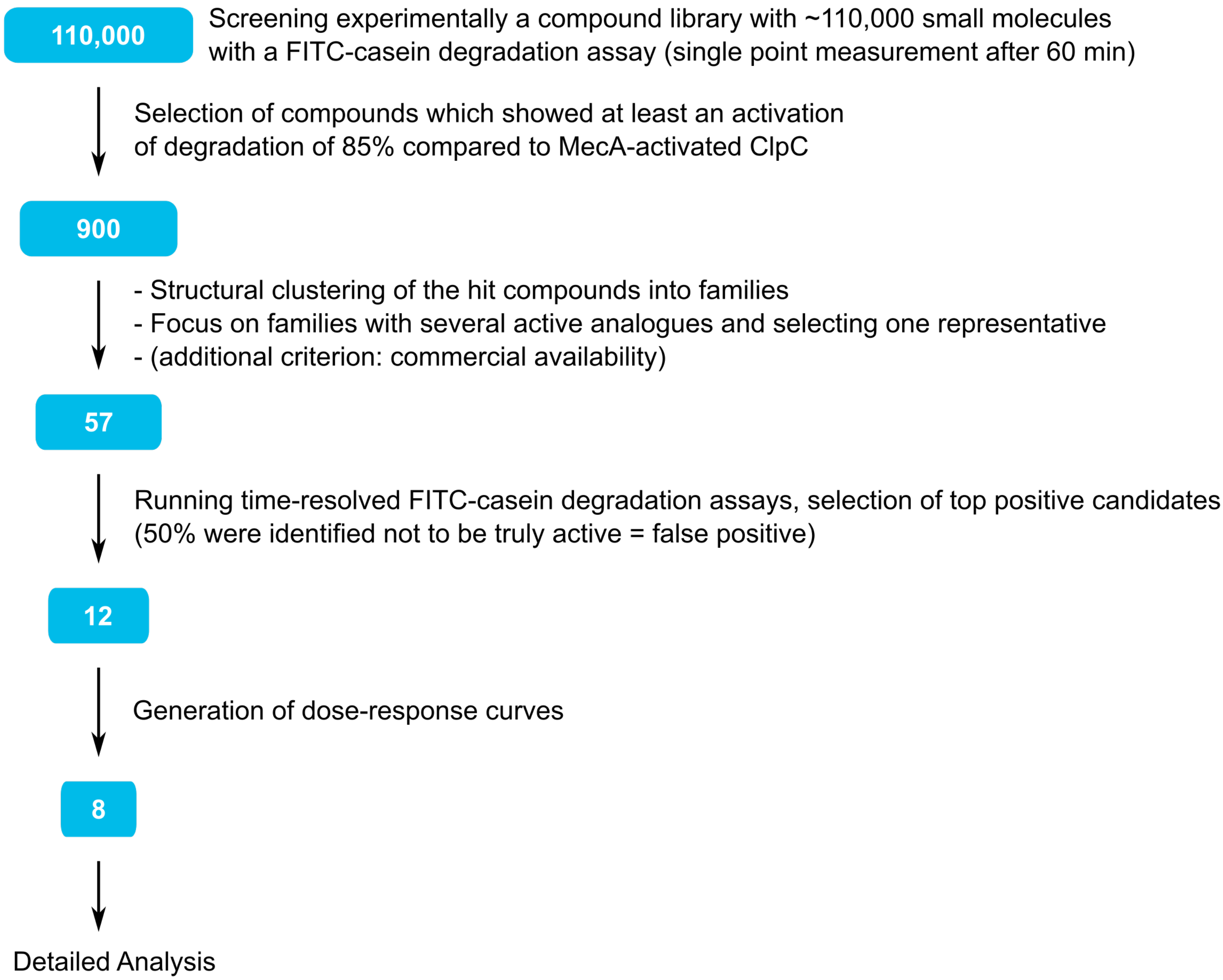
110,000
Screening experimentally a compound library with ~110,000 small molecules
with a FITC-casein degradation assay (single point measurement after 60 min)
Selection of compounds which showed at least an activation
of degradation of 85% compared to MecA-activated ClpC
900
- Structural clustering of the hit compounds into families
- Focus on families with several active analogues and selecting one representative
- (additional criterion: commercial availability)
57
Running time-resolved FITC-casein degradation assays, selection of top positive candidates
(50% were identified not to be truly active = false positive)
12
Generation of dose-response curves
8
Detailed Analysis

Figure S1

Figure S1

Flow chart of the process of identifying drug-like small molecules that enable efficient autonomous degradation of FITC-casein via ClpC/ClpP of *S. aureus.*

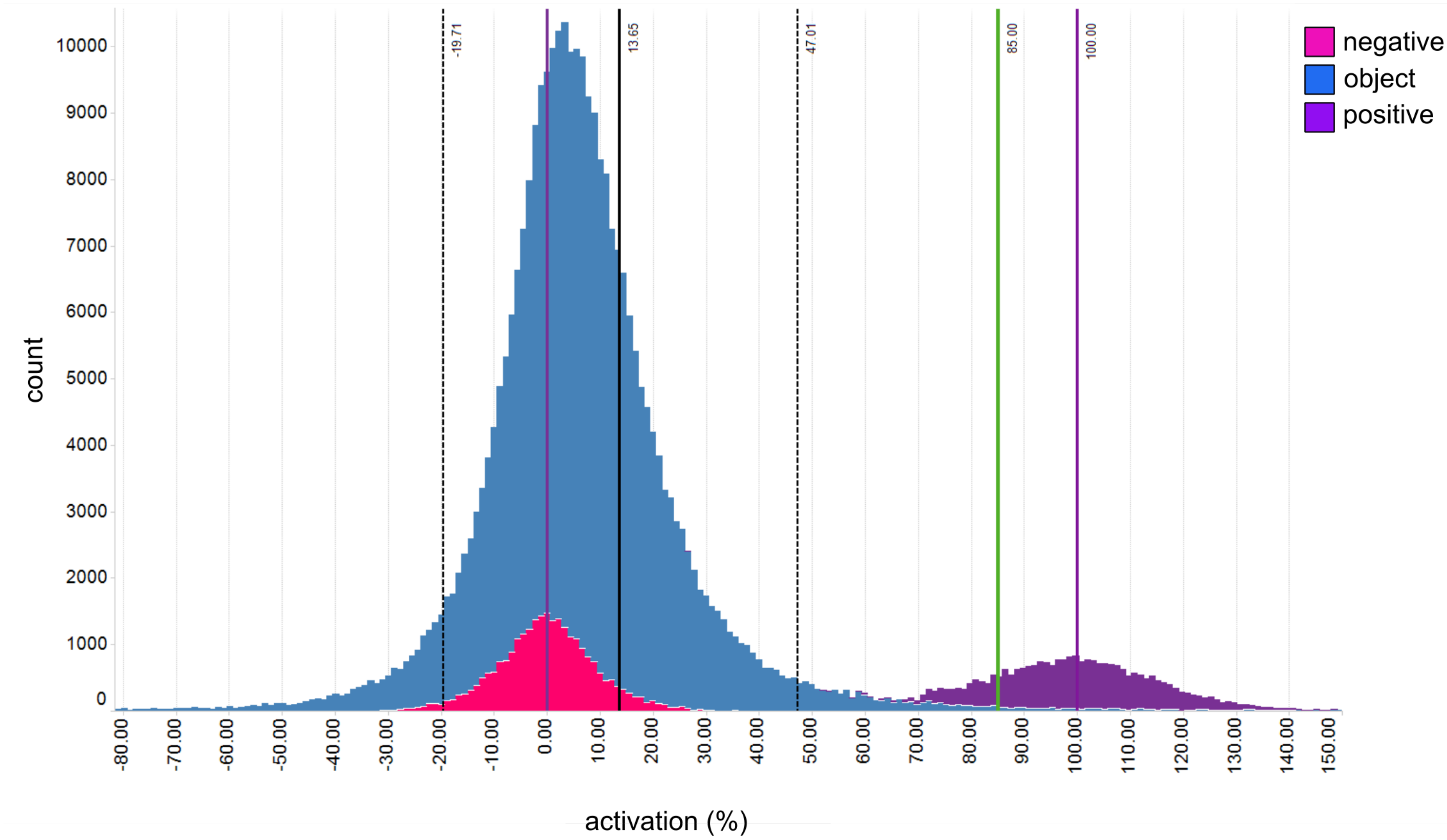

negative
object
positive
count
10000
9000
8000
7000
6000
5000
4000
3000
2000
1000
0
-19.71
13.65
47.01
85.00
100.00
-80.00
-70.00
-60.00
-50.00
-40.00
-30.00
-20.00
-10.00
0.00
10.00
20.00
30.00
40.00
50.00
60.00
70.00
80.00
90.00
100.00
110.00
120.00
130.00
140.00
150.00
activation (%)

Figure S2

Figure S2

Distribution of measured activity values for negative (magenta) and positive (purple) controls and screened compounds (objects - blue). Vertical lines in purple highlight in the histogram 0% and 100% activation. The black line shows the average activation value (13.65%), the dotted lines +/- one standard deviation of all screened compounds. The spread of the positive controls is relatively high but there is still good separation between the positive and negative controls. The green line at 85% highlights the applied cutoff for compound selection for the next filtering steps.

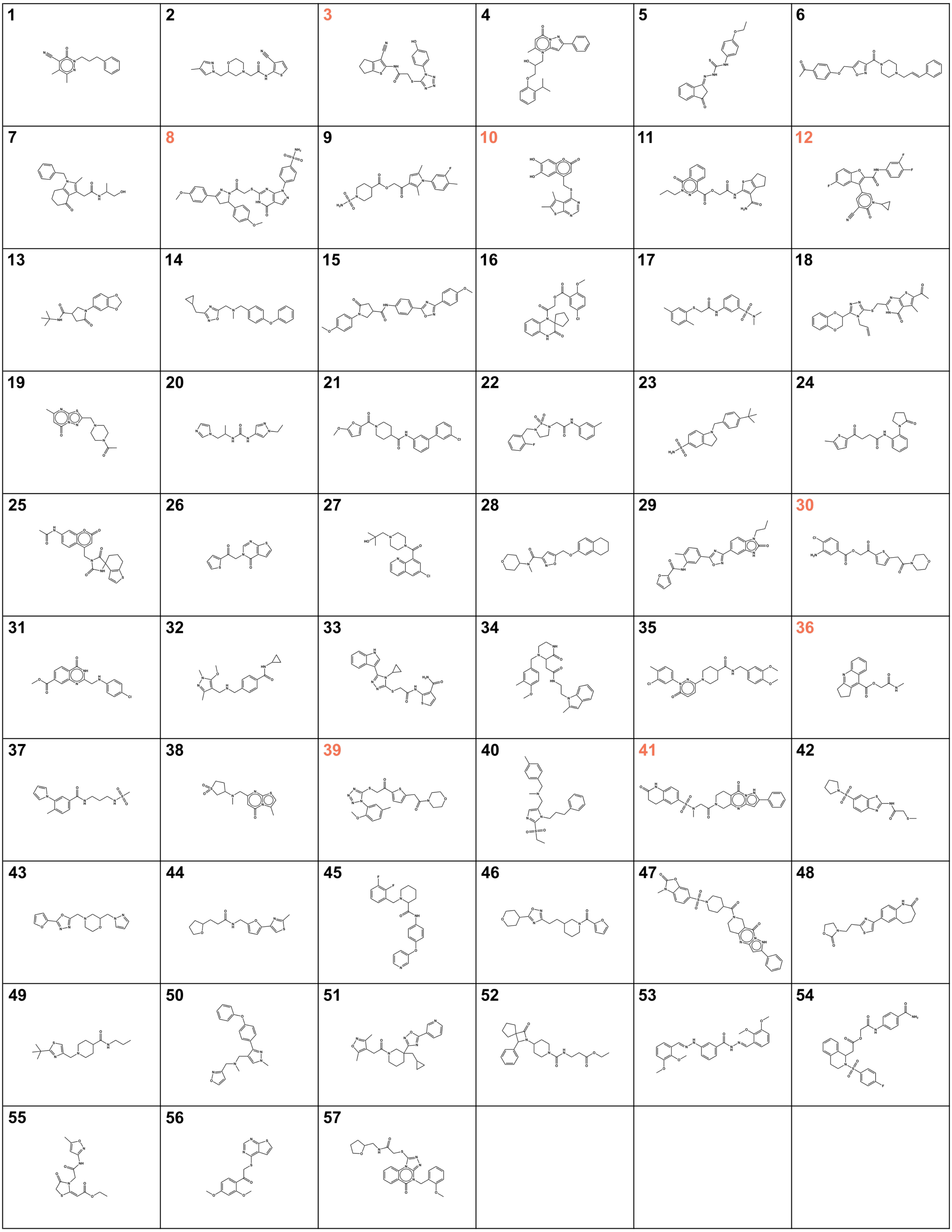
1
2
3
4
5
6
7
8
9
10
11
12
13
14
15
16
17
18
19
20
21
22
23
24
25
26
27
28
29
30
31
32
33
34
35
36
37
38
39
40
41
42
43
44
45
46
47
48
49
50
51
52
53
54
55
56
57

Figure S3

Figure S3

Chemical structures of identified primary hits. Compounds selected for extended characterization are labeled in red.

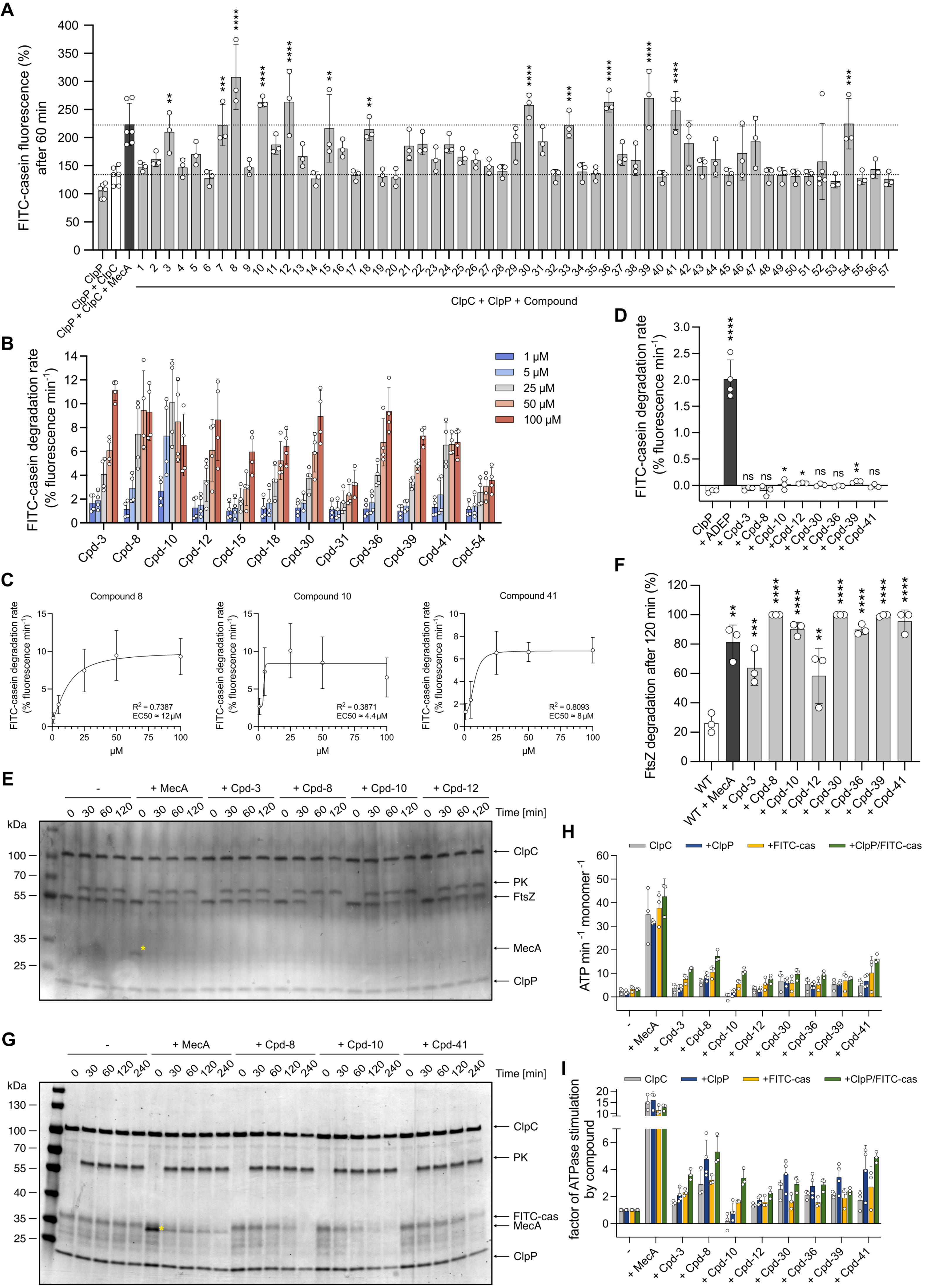
A
FITC-casein fluorescence (%) after 60 min
ClpP
ClpP + ClpC
ClpP + ClpC + MecA
ClpC + ClpP + Compound
B
FITC-casein degradation rate (% fluorescence min-1)
1 µM
5 µM
25 µM
50 µM
100 µM
Cpd-3
Cpd-8
Cpd-10
Cpd-12
Cpd-15
Cpd-18
Cpd-30
Cpd-31
Cpd-36
Cpd-39
Cpd-41
Cpd-54
C
Compound 8
Compound 10
Compound 41
R2 = 0.7387
EC50 ≈ 12µM
R2 = 0.3871
EC50 ≈ 4.4µM
R2 = 0.8093
EC50 ≈ 8µM
µM
D
ClpP
+ ADEP
ns
E
-
+ MecA
+ Cpd-3
+ Cpd-8
+ Cpd-10
+ Cpd-12
Time [min]
kDa
ClpC
PK
FtsZ
MecA
ClpP
F
FtsZ degradation after 120 min (%)
WT
WT + MecA
G
+ Cpd-41
FITC-cas
H
ClpC
+ClpP
+FITC-cas
+ClpP/FITC-cas
ATP min -1 monomer -1
I
factor of ATPase stimulation by compound

Figure S4

Figure S4

Identified and selected compounds specifically activate ClpC. (A) Total FITC-casein degradation activities after 60 min were determined in the presence of ClpP and ClpC + ClpP ± MecA or respective compounds (50 µM) Initial FITC-casein fluorescence was set to 100%. Data are represented as mean ±SD, n ≥ 3. (B-C) Dose-response curves of ClpC-WT were determined by monitoring FITC-casein degradation activities at increasing compound concentrations in the presence of ClpP (B). Afterwards, EC50 values were obtained by fitting agonist–response data with a four-parameter variable-slope nonlinear regression model (C). Data are represented as mean ±SD, n = 4. (D) FITC-casein degradation activities of ClpP were determined ± ADEP as positive control or respective compounds (50 µM). Data are represented as mean ±SD, n = 3. (E-F) FtsZ degradation activities were determined ± MecA or respective compounds (50 µM) in the presence of ClpP. A representative gel (E) for the quantification of the FtsZ degradation rates (see Figure 1D) and total FtsZ degradation after 120 min (F) is shown. Protein identities are indicated. The yellow asterisk marks the MecA band. PK = pyruvate kinase. (G) FITC-casein degradation activities were determined ± MecA or selected compounds (50 µM) in the presence of ClpP and indicated timepoints. Positions of proteins are indicated. (H-I) ATPase activities (H) and stimulation factors (I) of ClpC were determined under various conditions (alone, + ClpP, + FITC-casein, and ClpP + FITC-casein) ± MecA or respective compounds (50 µM). Data are represented as mean ±SD, n = 3.

Statistical significance was determined by one-way ANOVA with Dunnett's post-hoc test (D, F). Positive controls were assessed separately .ns = not significant, **$p < 0.01$, ***$p < 0.001$, ****$p < 0.0001$

A Cpd-8: 2D classes

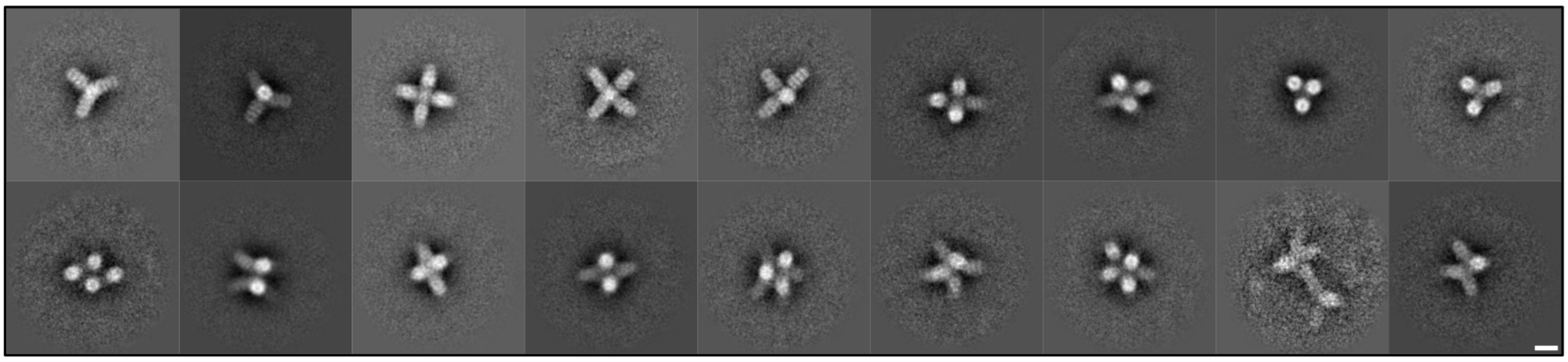

B Cpd-10: singe particles

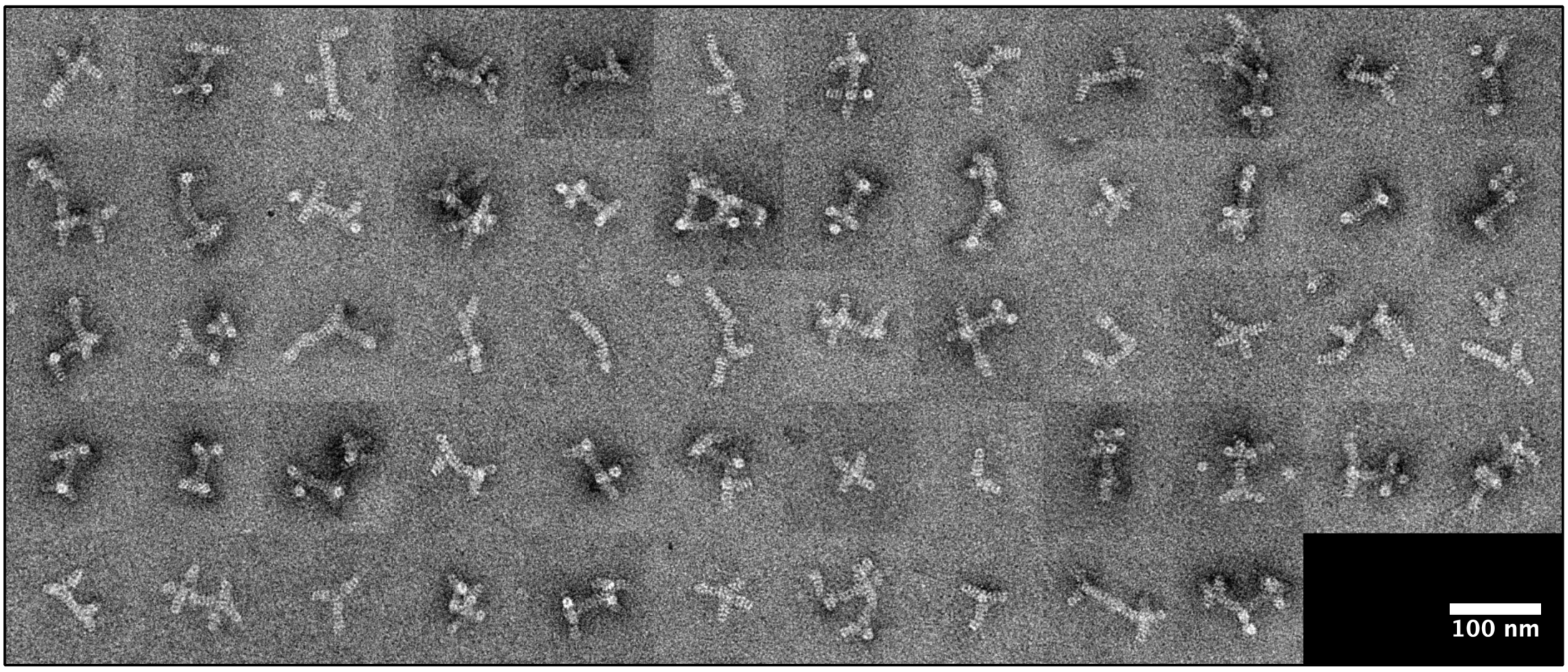

C Cpd-41: single particles

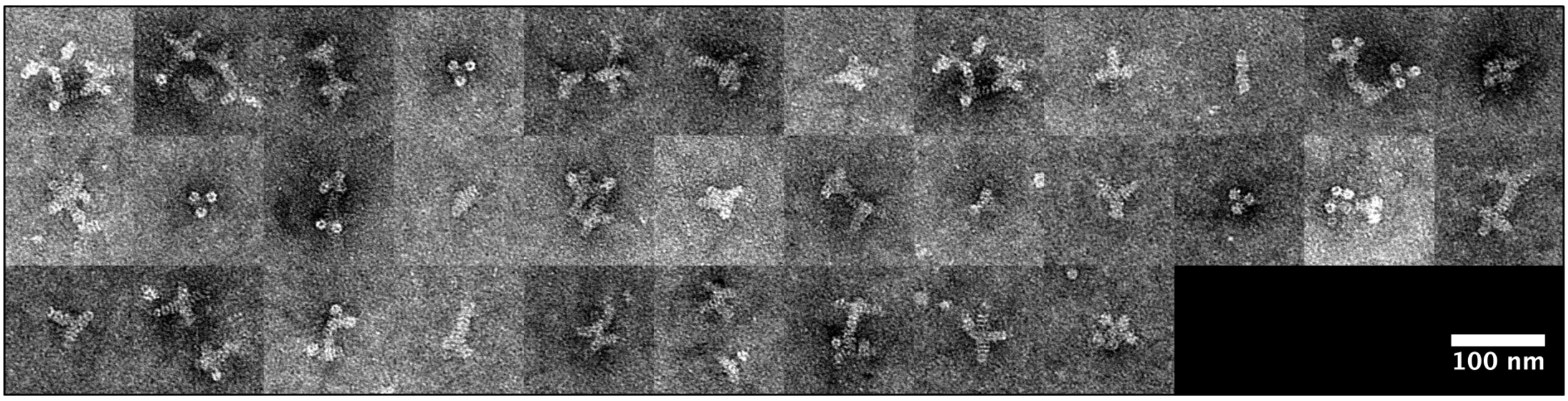

Figure S5

Figure S5

Compounds alter ClpC assembly states. (A) Additional 2D classes of Cpd-8 induced ClpC/ClpP assemblies as described in Figure 2C. Scale bar = 20 nm. (B-C) Representative single particles of ClpC/ClpP assemblies induced by Cpd-10 (B) and Cpd-41 (C) revealing high structural heterogeneity. Samples were subjected to EM following SEC as described in Figure 2C.

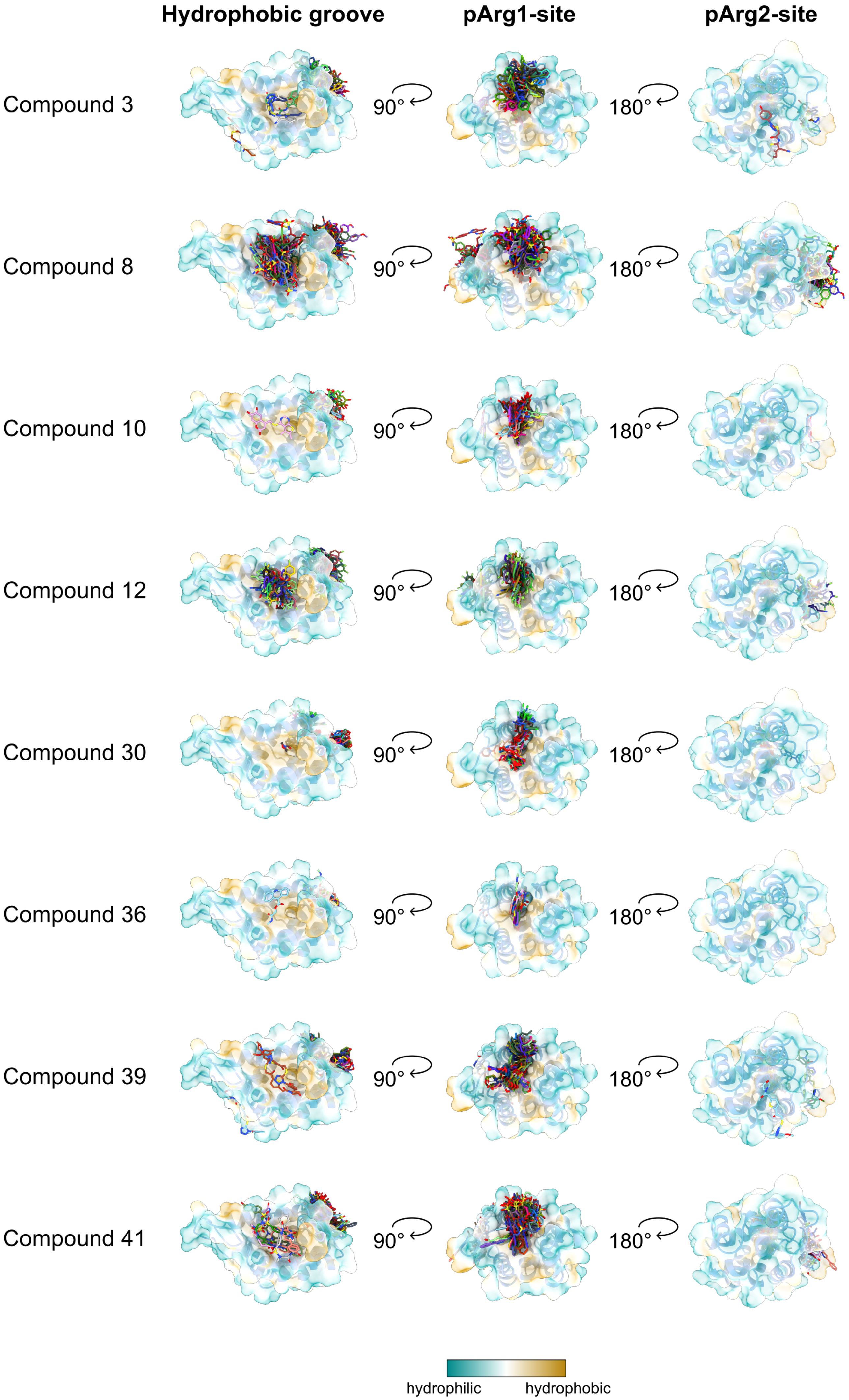

Hydrophobic groove
pArg1-site
pArg2-site
Compound 3
Compound 8
Compound 10
Compound 12
Compound 30
Compound 36
Compound 39
Compound 41
90°
180°
hydrophilic
hydrophobic

Figure S6

Figure S6

DiffDock simulations predict compounds to bind to the hydrophobic groove or to pArg1-/pArg2-sites of the NTD. DiffDock docking simulations (n = 40) of respective compounds to the ClpC N-terminal domain. The NTD is shown as a surface colored by hydrophobicity, with three orthogonal views highlighting the hydrophobic groove (left), the pArg1-site (middle), and the pArg2-site (right), with all predicted poses displayed for selected compounds.

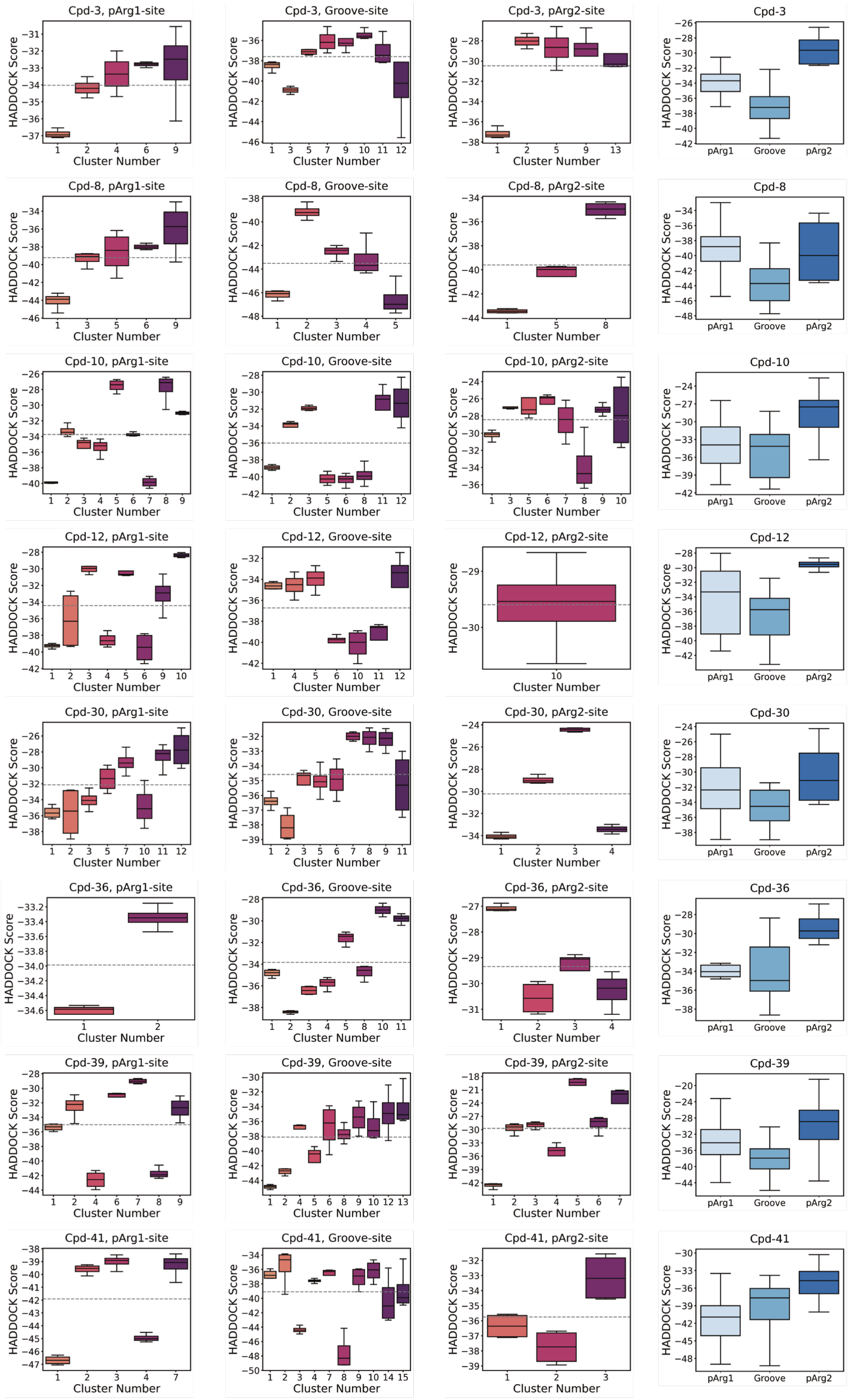

Cpd-3, pArg1-site
Cpd-3, Groove-site
Cpd-3, pArg2-site
Cpd-3
Cpd-8, pArg1-site
Cpd-8, Groove-site
Cpd-8, pArg2-site
Cpd-8
Cpd-10, pArg1-site
Cpd-10, Groove-site
Cpd-10, pArg2-site
Cpd-10
Cpd-12, pArg1-site
Cpd-12, Groove-site
Cpd-12, pArg2-site
Cpd-12
Cpd-30, pArg1-site
Cpd-30, Groove-site
Cpd-30, pArg2-site
Cpd-30
Cpd-36, pArg1-site
Cpd-36, Groove-site
Cpd-36, pArg2-site
Cpd-36
Cpd-39, pArg1-site
Cpd-39, Groove-site
Cpd-39, pArg2-site
Cpd-39
Cpd-41, pArg1-site
Cpd-41, Groove-site
Cpd-41, pArg2-site
Cpd-41
HADDOCK Score
Cluster Number
pArg1
Groove
pArg2

Figure S7

Figure S7

Total HADDOCK score for the corresponding compound docked to each of the indicated binding sites (pArg1, Hydrophobic groove, and pArg2). The grey dashed line indicates a mean across all clusters; the solid line in the boxes indicates the median, and the box outlines the 25th and 75th percentiles, respectively.

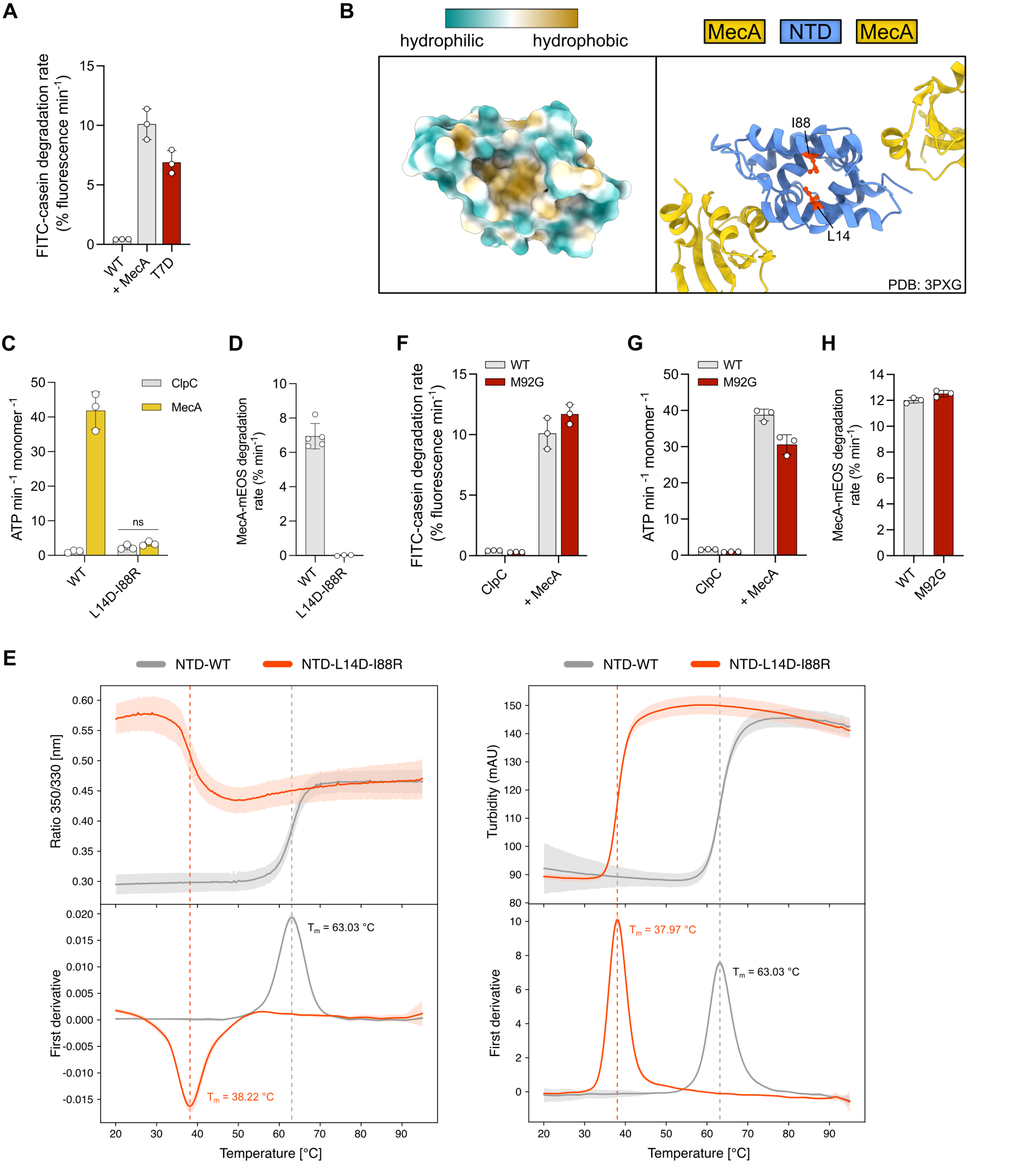

A
FITC-casein degradation rate (% fluorescence min-1)
WT
+ MecA
T7D
B
hydrophilic
hydrophobic
MecA
NTD
MecA
I88
L14
PDB: 3PXG
C
ATP min -1 monomer -1
ClpC
MecA
ns
WT
L14D-I88R
D
MecA-mEOS degradation rate (% min-1)
WT
L14D-I88R
F
FITC-casein degradation rate (% fluorescence min-1)
WT
M92G
ClpC
+ MecA
G
ATP min -1 monomer -1
WT
M92G
ClpC
+ MecA
H
MecA-mEOS degradation rate (% min-1)
WT
M92G
E
NTD-WT
NTD-L14D-I88R
Ratio 350/330 [nm]
First derivative
Tm = 63.03 °C
Tm = 38.22 °C
Temperature [°C]
Turbidity (mAU)
Tm = 37.97 °C
Tm = 63.03 °C

Figure S8

Figure S8

Mutation of the NTD hydrophobic groove can have destabilizing effects. (A) FITC-casein degradation activities of ClpC-WT ± MecA and ClpC-T7D in the presence of ClpP. Data are represented as mean ±SD, n = 3. (B) Structural representation of ClpC in complex with MecA (Wang et al. 2011), highlighting hydrophobicity of the ClpC-NTD (left) and residues subjected to mutagenesis located at the hydrophobic groove (L14, I88). (C) ATPase activities of ClpC-WT and ClpC-L14D-I88R were determined in presence of ClpP ± MecA. Data are represented as mean ±SD, n = 3. (D) MecA-mEOS degradation activities of ClpC-WT and ClpC-L14D-I88R were determined. Data are represented as mean ±SD, n ≥ 3. (E) The melting temperatures (T_m) of NTD-WT and NTD-L14D-I88R were determined via nanoDSF. First derivatives of the 350/330 nm ratio and sample turbidity were determined to calculate T_m. Data are represented as mean ±SD, n = 3. (F) FITC-casein degradation activities of ClpC-WT and ClpC-M92G ± MecA in the presence of ClpP. Data are represented as mean ±SD, n = 3. (G) ATPase activities of ClpC-WT and ClpC-M92G were determined ± MecA. Data are represented as mean ±SD, n = 3. (H) MecA-mEOS degradation activities of ClpC-WT and ClpC-M92G were determined. Data are represented as mean ±SD, n = 3.

ns = not significant by unpaired t-test (C).

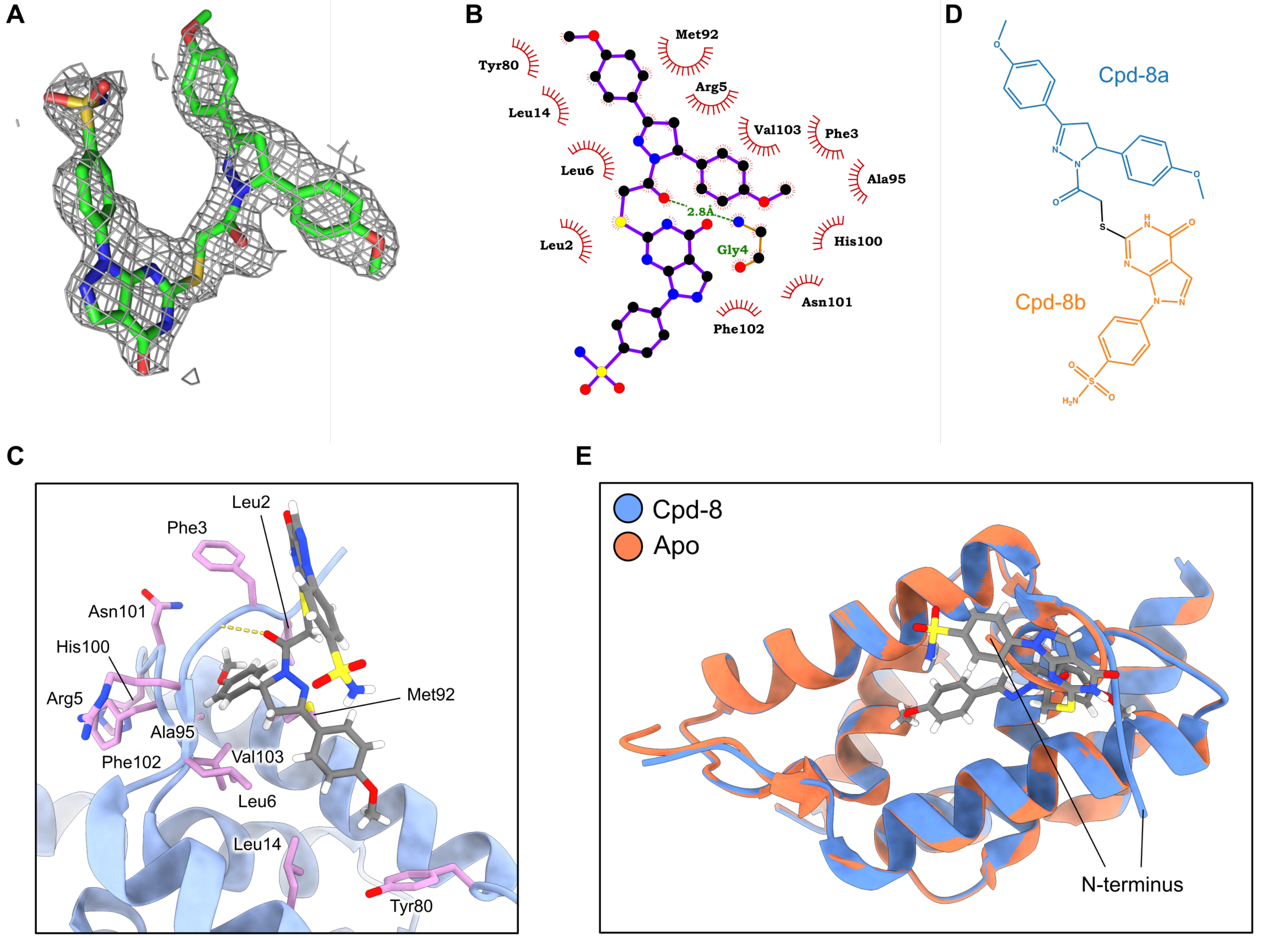
A
B
Tyr80
Met92
Leu14
Arg5
Val103
Phe3
Leu6
Ala95
2.8Å
Gly4
Leu2
His100
Asn101
Phe102
D
Cpd-8a
Cpd-8b
C
Leu2
Phe3
Asn101
His100
Arg5
Ala95
Phe102
Val103
Met92
Leu6
Leu14
Tyr80
E
Cpd-8
Apo
N-terminus

Figure S9

Figure S9

Crystal structure of the ClpC-NTD with bound Cpd-8. (A) Mesh representation (grey) of the $2mF_{obs}$-DF_{calc} electron density map around the bound ligand Cpd-8 (carbon atoms in green) at a contour level of 1 σ. (B) Schematic two-dimensional depiction of the interactions between ClpC-NTD and Cpd-8 created with LigPlot+ (Laskowski and Swindells 2011). The hydrogen bond between Gly4 and Cpd-8 is shown in green, and the residues forming hydrophobic contacts (cutoff ≤ 4Å) are shown as red partial circles. (C) Close-up view of Cpd-8 (carbons in grey) bound to ClpC-NTD (blue) with contacting residues (plum) and the hydrogen bond (yellow dashed line) between Gly4 and Cpd-8. (D) Cpd-8 can be subdivided in two parts: Cpd-8a (blue) and Cpd-8b (orange). (E) Superposition of ClpC-NTD apo (orange) with ClpC-NTD:Cpd-8 (blue/grey) shows the rearrangement of the N-terminus upon binding of Cpd-8.

A

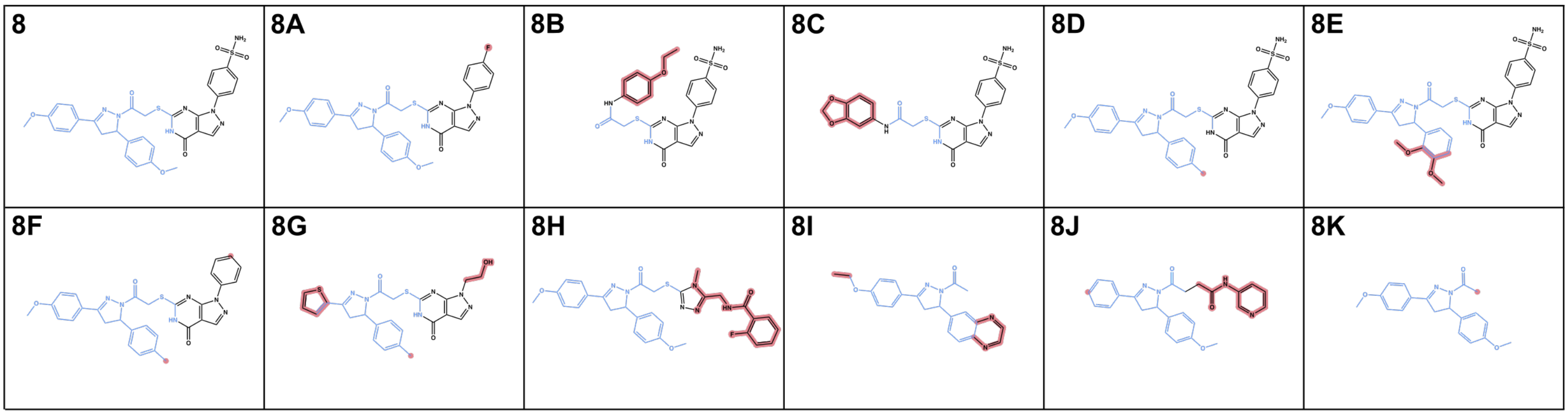

B

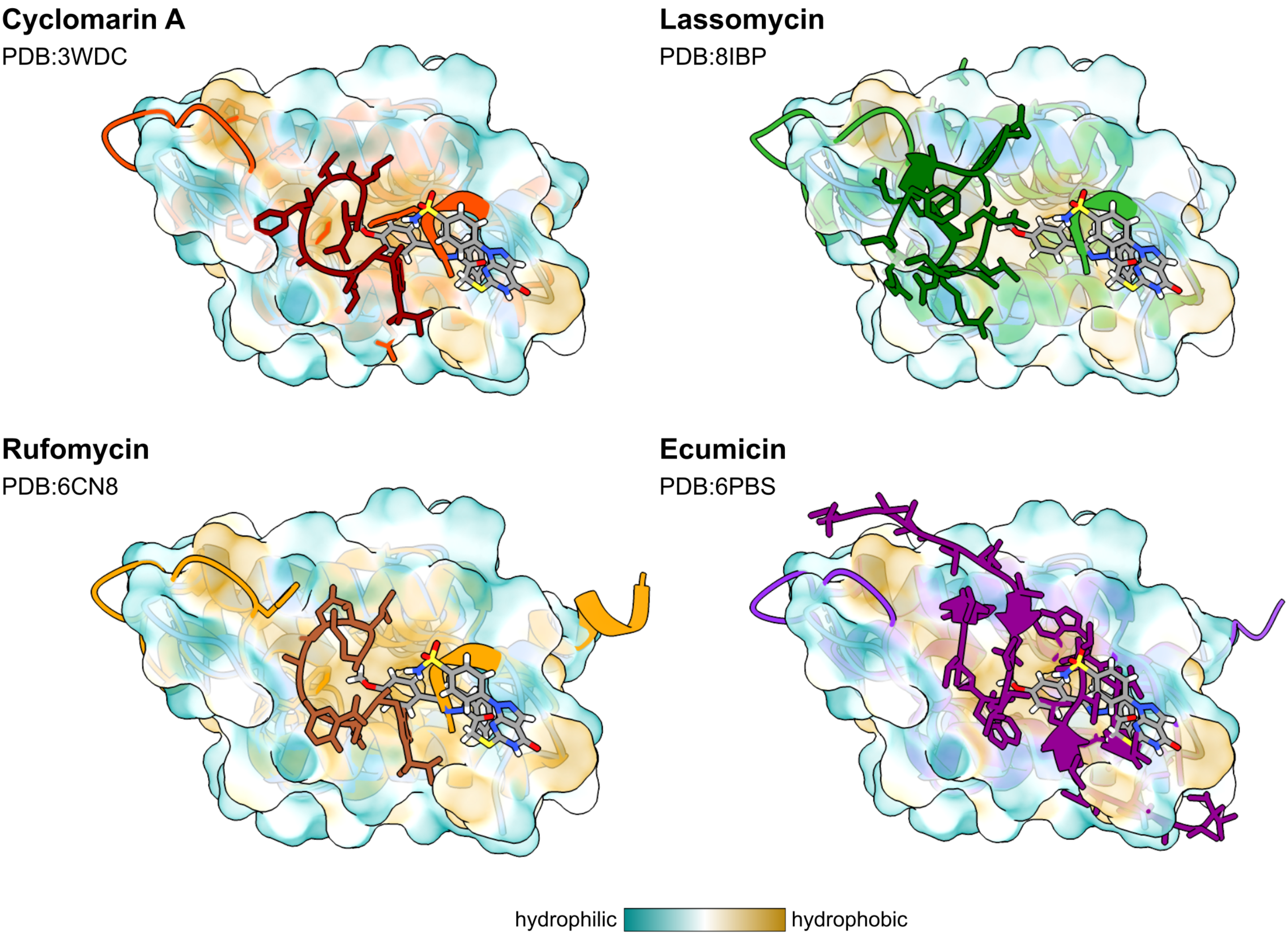

Figure S10

Figure S10

(A) Chemical structures of Cpd-8 and selected derivatives. Structural elements of Cpd-8 involved in protein–ligand contacts are displayed in blue. Modifications of respective derivatives are highlighted in red. (B) The Sa ClpC-NTD:Cpd-8 co-crystal structure was structurally aligned with available co-crystal structures of the Mtb ClpC1-NTD in complex with various cyclic peptides.

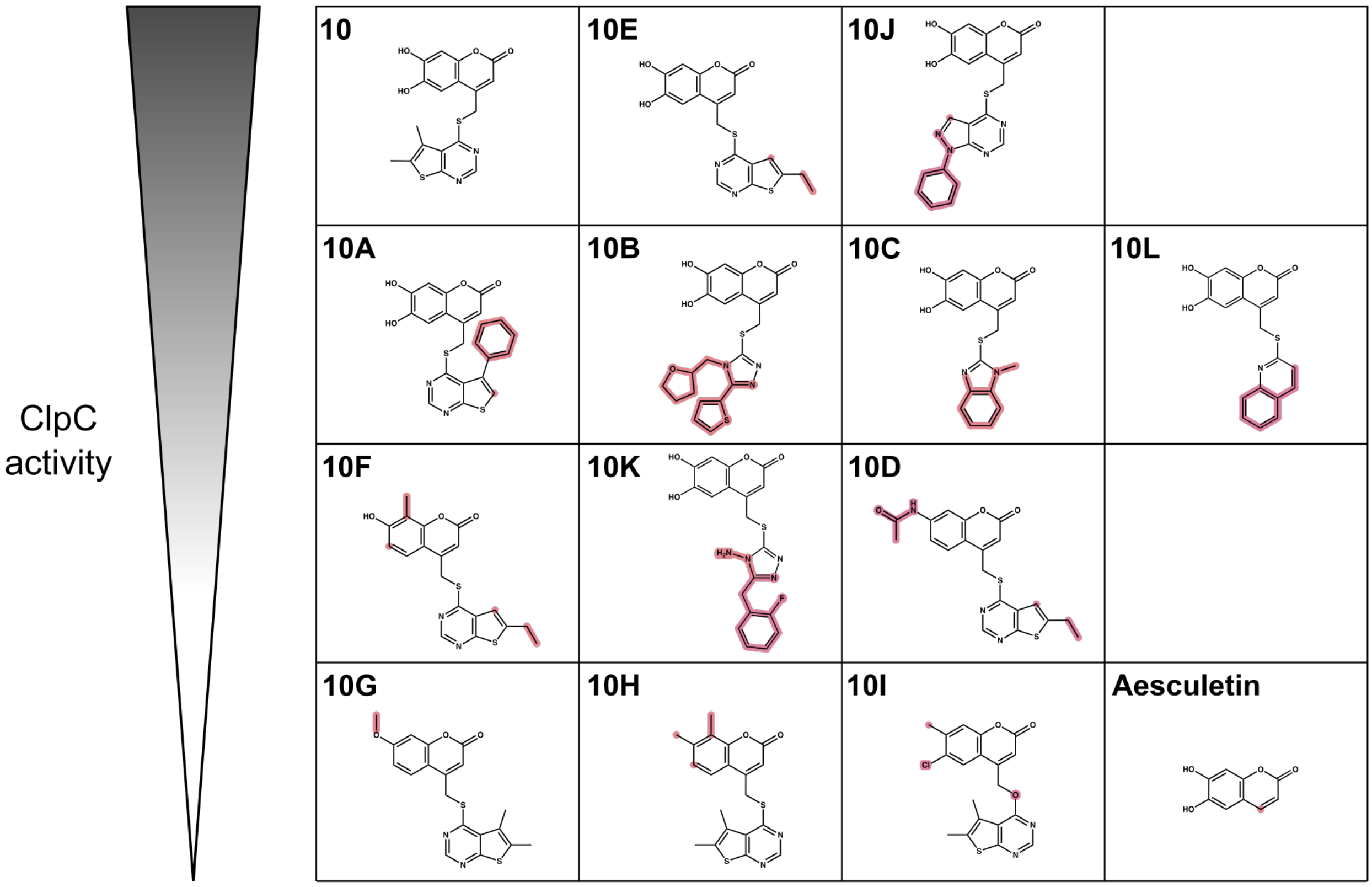
ClpC
activity
10
10E
10J
10A
10B
10C
10L
10F
10K
10D
10G
10H
10I
Aesculetin

Figure S11

Figure S11

Chemical structures of Cpd-10 and derivatives. Derivatives are roughly sorted by their stimulatory effects on ClpC/ClpP FITC-casein degradation activities (see Figure 6D). Modifications of respective derivatives are highlighted in red.

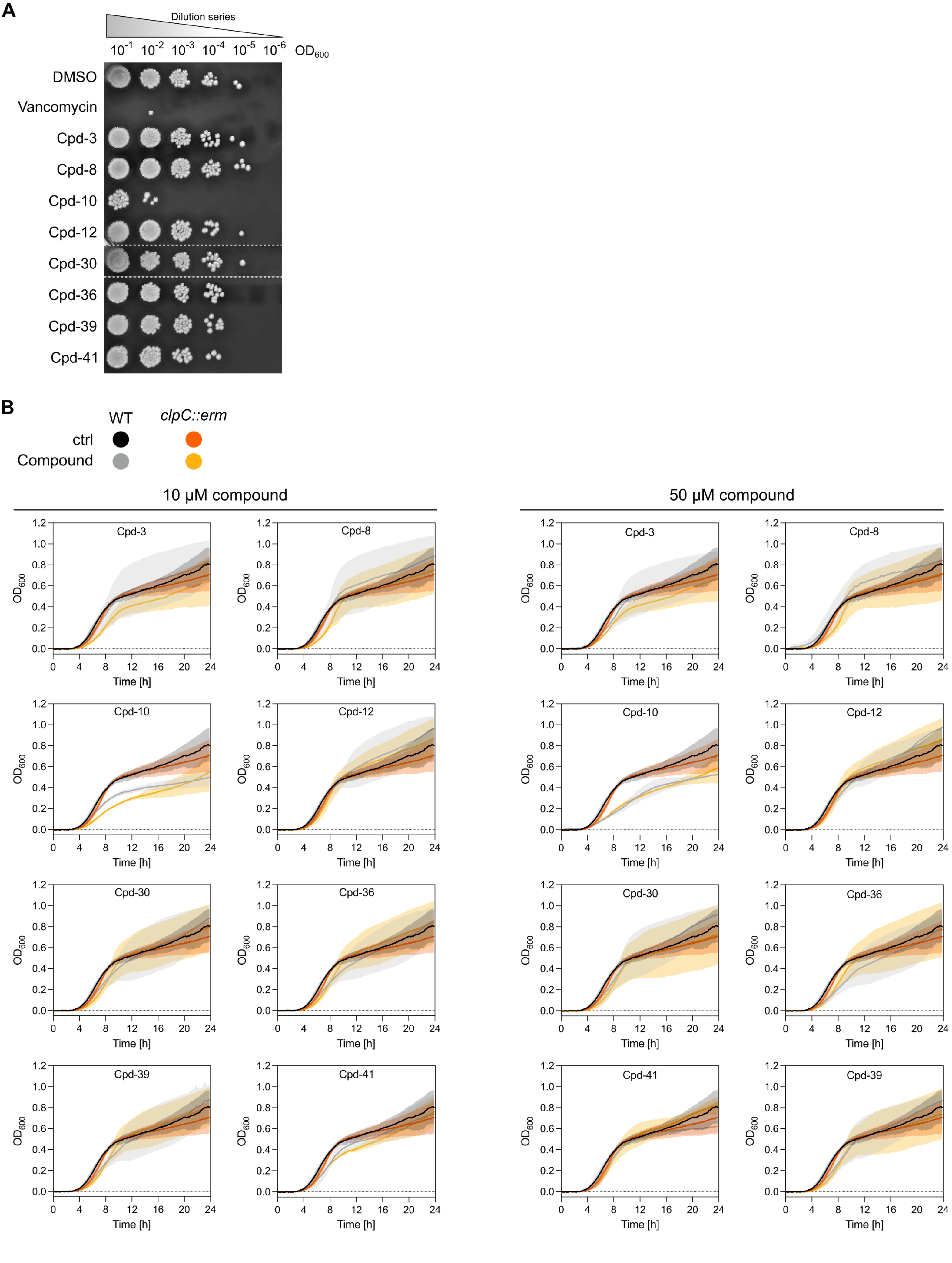
A
Dilution series
10^-1 10^-2 10^-3 10^-4 10^-5 10^-6 OD600
DMSO
Vancomycin
Cpd-3
Cpd-8
Cpd-10
Cpd-12
Cpd-30
Cpd-36
Cpd-39
Cpd-41
B
WT
clpC::erm
ctrl
Compound
10 µM compound
50 µM compound
Cpd-3
Cpd-8
Cpd-10
Cpd-12
Cpd-30
Cpd-36
Cpd-39
Cpd-41
OD600
Time [h]

Figure S12

Figure S12

Compounds induce ClpC/ClpP independent toxicity in *Staphylococcus aureus*. (A) Representative agar plate used to assess cell viability after compound treatment (see Figures 7A and 7B). (B) Growth curves of *S. aureus* WT and *clpC::erm* treated with respective compounds (10 & 50 µM) were recorded at 37°C for 24 h. Data are represented as mean ±SD, n = 3.

Table S1: Data collection and refinement statistics for the N-terminal domain of S. aureus ClpC-NTD apo and with bound ligand cpd8. Data for highest-resolution shell are in parentheses.

	**ClpC-NTD apo**	**ClpC-NTD:Cpd8**
**Data collection**		
Beamline, wavelength (Å)	ESRF ID30-A1, 0.9677	ESRF ID30-A1, 0.9677
Space group	$P4_32_12$	$P4_32_12$
Resolution range (Å)	38.47-1.78 (1.82-1.78)	39.51-1.90 (1.94-1.90)
Total reflections	435570 (25508)	248442 (16388)
Unique reflections	18608 (1031)	15514 (956)
a, b, c (Å)	60.86, 60.86, 99.29	60.75, 60.75, 100.63
α, β, γ (°)	90, 90, 90	90, 90, 90
R_{merge}	0.098 (2.938)	0.105 (2.852)
R_{pim}	0.021 (0.611)	0.028 (0.726)
Mean ($I/\sigma(I)$)	18.2 (1.2)	14.9 (1.2)
Multiplicity	23.4 (24.1)	16.0 (17.1)
Completeness (%)	100 (100)	100 (100)
$CC_{1/2}$	0.999 (0.617)	0.999 (0.472)
**Refinement**		
R_{work}	0.1911 (0.3716)	0.1946 (0.2723)
R_{free}	0.2207 (0.3763)	0.2141 (0.2853)
RMSD bond length (Å)	0.006	0.006
RMSD bond angle (°)	0.700	0.730
Ramachandran favored (%)	98.51	99.24
Ramachandran allowed (%)	1.49	0.76
Ramachandran outliers (%)	0	0
Rotamer outliers (%)	0	0
Clashscore	0.88	0.85
Average B-factor ($Å^2$)	37.6	41.2
Protein	37.2	40.4
Ligands	47.3	53.7
Solvent	40.5	39.8
**PDB identifier**	9TXC	9TXD

Table S2: Strains and plasmids used in this study

Strain	Description	Source or reference
E. coli XL1 blue	*recA1 endA1 gyrA96 thi-1 hsdR1 supE44 relA1 lac* [F' *proAB lacI^q^ ΔM15* Tn10 (Tcr)]	Stratagene
E. coli BL21	*F- ompT lon hsdSB gal dcm λ* (DE3)	Novagen
E. coli ΔclpB	MC4100 Δ*clpB* (Kanamycin sensitive)	(1)
**Plasmid**	**Description**	**Source or reference**
pDS56-*clpC*	Vector for IPTG-inducible expression of ClpC-WT in *E. coli ΔclpB* cells	(2)
pDS56-*clpP*	Vector for IPTG-inducible expression of ClpP in *E. coli ΔclpB* cells	(2)
pDS56-*mecA*	Vector for IPTG-inducible expression of MecA in *E. coli ΔclpB* cells	(2)
pDS56-*clpC*-ΔN	Vector for IPTG-inducible expression of ClpC-ΔN (2-146) in *E. coli ΔclpB* cells	(2)
pDS56-*clpC*-ΔN-F436A	Vector for IPTG-inducible expression of ClpC-ΔN-F436A in *E. coli ΔclpB* cells	(2)
pDS56-*clpC*-E280A/E618A (DWB)	Vector for IPTG-inducible expression of ClpC-DWB (E280A/E618A) in *E. coli ΔclpB* cells	(2)
pDS56-*clpC*-T7D	Vector for IPTG-inducible expression of ClpC-T7D in *E. coli ΔclpB* cells	(1)
pDS56-*clpC*-M92G	Vector for IPTG-inducible expression of ClpC-M92G in *E. coli ΔclpB* cells	This study
pDS56-*clpC*-L14D-I88R	Vector for IPTG-inducible expression of ClpC-L14D-I88R in *E. coli ΔclpB* cells	This study
pET24a-*clpC*-NTD (1-155)	Vector for IPTG-inducible expression of ClpC-NTD-WT in *E. coli* BL21 cells	This study
pET24a-*clpC*-NTD-L14D-I88R (1-155)	Vector for IPTG-inducible expression of ClpC-NTD-L14D-I88R in *E. coli* BL21 cells	This study
pET24a-ftsZ	Vector for IPTG-inducible expression of FtsZ in *E. coli* BL21 cells	(3)
pDS56-*mecA-mEOS3.2*	Vector for IPTG-inducible expression of MecA-mEOS3.2 in *E. coli ΔclpB* cells	(4)

Table S3: Primers for sequencing, mutagenesis, and restriction cloning used in this study. Lowercase letters indicate mutated nucleotides.

Primer	Sequence
pDS56_fw_seq	CTTTGTCTTCACCTCGAG
SaClpC_400-bp_seq	CGTGCACAAGTTGTGAAAG
SaClpC_1000-bp_seq	GTTTCCAACCTGTACAAG
SaClpC_1600-bp_seq	CTAAACGACCAATTGGTAGC
SaClpC_2000-bp_seq	CAACGATTTGCTGGATTC
SaClpC_L14D_fw	GCACAGCGCGTAgatGCACATGC
SaClpC_L14D_rv	GCATGTGCatcTACGCGCTGTGC
SaClpC_I88R_fw	CCTAGAGCTAAAAAAGTCAggGAATTATCGATGGATGAAGC
SaClpC_I88R_rv	GCTTCATCCATCGATAATTCccTGACTTTTTTAGCTCTAGG
SaClpC_M92A_rv	[Phos]ACTTTTTTAGCTCTAGGTGTATAATGCAATGTACCAAC
SaClpC_M92G_fw	[Phos]CATTGAATTATCGggaGATGAAGCTAGAAATTAC
SaClpC_NTD_NdeI	ggccatcatatgTTATTTGGTAGATTAACTGAGCGTGC
SaClpC_NTD_XhoI	ggccatctcgagACTAGCTTGTGCATTTTTATTACTCAT

Table S4: List of used compounds in this study.

Compound	SMILES	Supplier	Supplier-ID
1	C1(=C(C(=NN(C1=O)CCCc2ccccc2)C)C)C#N	ENAMINE	Z98425649
2	c1(c(ccs1)C#N)NC(=O)CN3CC(Cn2ncc(c2)C)OCC3	ENAMINE	Z1139494673
3	c1(c(c2c(s1)CCC2)C#N)NC(=O)CSc3n(nnn3)-c4ccc(cc4)O	ENAMINE	Z25096565
4	c31n(nc(c1)-c2ccccc2)C(=O)C=C(N3CC(COc4c(cccc4)C(C)C)O)C	ENAMINE	Z317187974
5	C3(=NNC(=S)Nc1ccc(cc1)OCC)c2c(cccc2)C(=O)C3	ENAMINE	Z56836505
6	c1(noc(c1)COc2ccc(cc2)C(=O)C)C(=O)N3CCN(CC3)CC=Cc4ccccc4	ChemBridge	14362104
7	n1(c2c(c(c1C)CC(=O)NC(CO)C)C(=O)CCC2)Cc3ccccc3	ChemBridge	11469948
8	c31c(cnn1-c2ccc(cc2)S(=O)(=O)N)C(=O)NC(=N3)SCC(=O)N4N=C(CC4c5ccc(cc5)OC)c6ccc(cc6)OC	ChemDiv	C099-0754
9	n1(c(c(cc1C)C(=O)COC(=O)C2CCN(S(=O)(=O)N)CC2)C)-c3cc(c(cc3)C)F	ENAMINE	Z324869730
10	c21c(c(c(s1)C)C)c(ncn2)SCC3=CC(=O)Oc4c3cc(c(c4)O)O	ENAMINE	Z20229898
11	c1(c(c2c(s1)CCC2)C(=O)N)NC(=O)COC(=O)C3=NN(C(=O)c4c3cccc4)CCC	ENAMINE	Z15814770
12	c1(c(c2c(o1)ccc(c2)F)C3=CN(C(=O)C(=C3)C#N)C4CC4)C(=O)Nc5cc(c(cc5)F)F	ENAMINE	Z381676984
13	N1(C(=O)CC(C(=O)NC(C)(C)C)C1)c2cc3c(cc2)OCO3	ENAMINE	Z339436122
14	n1c(onc1CC2CC2)CN(Cc3ccc(cc3)Oc4ccccc4)C	ChemBridge	24881321
15	n1c(noc1-c2ccc(cc2)NC(=O)C3CN(C(=O)C3)c4ccc(cc4)OC)-c5ccc(cc5)OC	ChemDiv	D400-2814
16	N1(C3(C(=O)Nc2c1cccc2)CCCC3)C(=O)COC(=O)c4c(ccc(c4)Cl)OC	ENAMINE	Z193532094
17	S(=O)(=O)(c1cc(ccc1)NC(=O)CSc2c(ccc(c2)C)C)N(C)C	ENAMINE	Z25046369
18	c21c(sc(c1C)C(=O)C)N=C(NC2=O)CSc3n(c(nn3)C5Oc4c(cccc4)OC5)CC=C	ENAMINE	Z24181224
19	N21C(=NC(=CC1=O)C)SC(=N2)CN3CCN(C(=O)C)CC3	ENAMINE	Z123748562
20	c1(cn(nc1)CC)NC(=O)NC(Cn2cncc2)C	ENAMINE	Z552040506
21	c1(oc(cc1)OC)C(=O)N4CCC(C(=O)Nc2cc(ccc2)-c3cc(ccc3)Cl)CC4	ChemBridge	24913716
22	S2(=O)(=O)N(CC(=O)Nc1cc(ccc1)C)CCN2Cc3c(cccc3)F	ChemDiv	E870-0226
23	S(=O)(=O)(c1cc3c(cc1)N(Cc2ccc(cc2)C(C)(C)C)CC3)N	ENAMINE	Z195929716
24	N3(c1c(cccc1)NC(=O)CCC(=O)c2sc(cc2)C)C(=O)CCC3	ENAMINE	Z109734790
25	N1(C(=O)C3(NC1=O)c2c(scc2)CCC3)CC4=CC(=O)Oc5c4ccc(c5)NC(=O)C	ENAMINE	Z17375748
26	c21c(scc1)N=CN(C2=O)CC(=O)c3sccc3	ENAMINE	Z88068784
27	c1(c2c(cc(c1)Cl)cccn2)C(=O)N3CCN(CC(O)(C)C)CC3	ENAMINE	Z1143012004
28	c1(noc(c1)COc2cc3c(cc2)CCCC3)C(=O)N(C4CCOCC4)C	ChemBridge	25822347
29	C5(=O)N(c1c(cc(cc1)-c2nc(on2)-c3cc(c(cc3)C)NC(=O)c4occc4)N5)CCC	ChemDiv	F543-0446
30	c1(sc(cc1)CC(=O)N2CCOCC2)C(=O)COC(=O)c3cc(c(cc3)Cl)N	ENAMINE	Z87542916

31	C2(=O)c1c(cc(cc1)C(=O)OC)N=C(N2)CNc3ccc(cc3)Cl	ENAMINE	Z44498285
32	c1(c(n(nc1C)C)OC)CNCc2ccc(cc2)C(=O)NC3CC3	ENAMINE	Z991043786
33	c1(n(c(nn1)SCC(=O)Nc2c(ccs2)C(=O)N)C3CC3)-c4c[nH]c5c4cccc5	ENAMINE	Z92491919
34	n1(c(cc2c1cccc2)C)CCNC(=O)CC4N(Cc3cc(c(cc3)OC)C)CCNC4=O	ChemBridge	26830763
35	N3=C(N2CCC(C(=O)NCc1cc(c(cc1)OC)OC)CC2)C=CC(=O)N3c4cc(c(cc4)C)Cl	ChemDiv	F699-0948
36	c1(c3c(nc2c1cccc2)CCC3)C(=O)OCC(=O)NC	ENAMINE	Z19180215
37	S(=O)(=O)(NCCCNC(=O)c1cc(c(cc1)C)-n2cccc2)C	ENAMINE	Z318215160
38	C21=NC(=CC(=O)N1C(=CS2)C)CN(C3CS(=O)(=O)CC3)C	ENAMINE	Z64875178
39	n1(c(nnn1)SCC(=O)c2sc(cc2)CC(=O)N3CCOCC3)-c4c(ccc(c4)C)OC	ENAMINE	Z87696135
40	c1(n(c(cn1)CN(Cc2ccc(cc2)C)C)CCCc3ccccc3)S(=O)(=O)CC	ChemBridge	44641602
41	N51C(=NC4=C(C1=O)CCN(C(=O)CN(S(=O)(=O)c2cc3c(cc2)NC(=O)CC3)C)C4)C=C(N5)c6ccccc6	ChemDiv	S021-2319
42	S(=O)(=O)(c2cc1sc(nc1cc2)NC(=O)CSC)N3CCCC3	ENAMINE	Z368037132
43	c1(nnc(o1)CN3CC(Cn2nccc2)OCC3)-c4occc4	ENAMINE	Z1139493323
44	c1(nc(sc1)C)-c2oc(cc2)CNC(=O)CCC3OCCC3	ENAMINE	Z363503400
45	N3(C(C(=O)Nc1ccc(cc1)Oc2cnccc2)CCCC3)Cc4c(c(ccc4)F)F	ChemBridge	45509512
46	n1c(onc1CCC3CN(C(=O)c2occc2)CCC3)C4CCOCC4	ChemDiv	SA66-1053
47	N61C(=NC5=C(C1=O)CN(C(=O)C4CCN(S(=O)(=O)c2cc3c(cc2)N(C(=O)O3)C)CC4)CC5)C=C(N6)c7ccccc7	ChemDiv	S022-1763
48	n1c(csc1CCN2C(=O)OCC2)-c3cc4c(cc3)NC(=O)CCC4	ENAMINE	Z735540942
49	n1c(scc1CN2CCC(C(=O)NCCC)CC2)C(C)(C)C	ENAMINE	Z382749092
50	c1(c(nn(c1C)C)-c2ccc(cc2)Oc3ccccc3)CN(Cc4nocc4)C	ChemBridge	58793950
51	n1c(noc1-c2cnccc2)C4(CN(C(=O)Cc3c(onc3C)C)CCC4)CC5CC5	ChemDiv	SA71-0459
52	C1(=O)C4(C(N1C2CCN(C(=O)NCCC(=O)OCC)CC2)c3ccccc3)CCCC4	ChemDiv	S631-0088
53	C(=O)(c1cc(ccc1)NN=Cc2c(c(ccc2)OC)OC)NN=Cc3c(c(ccc3)OC)OC	ENAMINE	Z44831364
54	S(=O)(=O)(N3C(CC(=O)OCC(=O)Nc1ccc(cc1)C(=O)N)c2c(cccc2)CC3)c4ccc(cc4)F	ENAMINE	Z56814472
55	N1(C(=CC(=O)OCC)SCC1=O)CC(=O)Nc2noc(c2)C	ENAMINE	Z25936859
56	c31c(ncnc1SCC(=O)c2c(cc(cc2)OC)OC)scc3	ENAMINE	Z20247185
57	c41n(c(nn1)SCC(=O)NCC2OCCC2)-c3c(cccc3)C(=O)N4Cc5c(cccc5)OC	ENAMINE	Z24927311
8A	O=C1C2=C(N(C3=CC=C(F)C=C3)N=C2)N=C(SCC(N(C(C4=CC=C(OC)C=C4)C5)N=C5C6=CC=C(OC)C=C6)=O)N1	ChemDiv	C099-0367
8B	O=S(C(C=C1)=CC=C1N(N=C2)C(N=C(SCC(NC(C=C3)=CC=C3OCC)=O)N4)=C2C4=O)(N)=O	ChemDiv	C099-0729
8C	O=S(C(C=C1)=CC=C1N(N=C2)C(N=C(SCC(NC3=CC(OCO4)=C4C=C3)=O)N5)=C2C5=O)(N)=O	ChemDiv	C099-0735
8D	O=S(C(C=C1)=CC=C1N(N=C2)C(N=C(SCC(N(C(C3=CC=C(C)C=C3)C4)N=C4C5=CC=C(OC)C=C5)=O)N6)=C2C6=O)(N)=O	ChemDiv	C099-0749
8E	O=S(C(C=C1)=CC=C1N(N=C2)C(N=C(SCC(N(C(C3)C4=CC=CC(OC)=C4OC)N=C3C5=CC=C(OC)C=C5)=O)N6)=C2C6=O)(N)=O	ChemDiv	C099-0756
8F	O=C1C2=C(N(C3=CC=CC=C3)N=C2)N=C(SCC(N(C(C4=CC=C(C)C=C4)C5)N=C5C6=CC=C(OC)C=C6)=O)N1	ChemDiv	C365-0069

8G	O=C1C2=C(N(CCO)N=C2)N=C(SCC(N(C(C(C=C3)=CC=C3C)C4)N=C4C5=CC=CS5)=O)N1	ChemDiv	F248-0084
8H	CN1C(SCC(N2C(C3=CC=C(OC)C=C3)CC(C4=CC=C(OC)C=C4)=N2)=O)=NN=C1CNC(C5=C(F)C=CC=C5)=O	Chemdiv	A0070264
8I	O=C(C)N1N=C(C2=CC=C(C=C2)OCC)CC1C3=CC=C4C(N=CC=N4)=C3	Enamine	Z355444676
8J	COC1=CC=C(C2CC(C3=CC=CC=C3)=NN2C(CCC(NC4=CC=CN=C4)=O)=O)C=C1	Enamine	Z52061882
8K	COC1=CC=C(C2CC(C3=CC=C(C=C3)OC)=NN2C(C)=O)C=C1	Enamine	Z166630838
10A	OC=1C=C2OC(=O)C=C(CSC=3N=CN=C4SC=C(C=5C=CC=CC5)C34)C2=CC1O	Enamine	Z20245441
10B	OC=1C=C2OC(=O)C=C(CSC3=NN=C(C4=CC=CS4)N3CC5CCCO5)C2=CC1O	Enamine	Z19524055
10C	CN1C(SCC2=CC(=O)OC=3C=C(O)C(O)=CC23)=NC=4C=CC=CC14	Enamine	Z15478450
10D	CCC1=CC=2C(SCC3=CC(=O)OC=4C=C(NC(=O)C)C=CC34)=NC=NC2S1	Enamine	Z13702705
10E	CCC1=CC=2C(SCC3=CC(=O)OC=4C=C(O)C(O)=CC34)=NC=NC2S1	Enamine	Z13702296
10F	CCC1=CC=2C(SCC3=CC(=O)OC=4C(C)=C(O)C=CC34)=NC=NC2S1	Enamine	Z13697652
10G	COC=1C=CC=2C(CSC=3N=CN=C4SC(C)=C(C)C34)=CC(=O)OC2C1	Enamine	Z20229746
10H	CC=1SC=2N=CN=C(SCC3=CC(=O)OC=4C(C)=C(C)C=CC34)C2C1C	Enamine	Z20229853
10I	CC=1SC=2N=CN=C(OCC3=CC(=O)OC=4C=C(C)C(Cl)=CC34)C2C1C	Enamine	Z16518499
10J	OC=1C=C2OC(=O)C=C(CSC=3N=CN=C4N(N=CC34)C=5C=CC=CC5)C2=CC1O	Enamine	Z16382406
10K	NN1C(CC=2C=CC=CC2F)=NN=C1SCC3=CC(=O)OC=4C=C(O)C(O)=CC34	Enamine	Z54913878
10L	OC=1C=C2OC(=O)C=C(CSC=3C=CC=4C=CC=CC4N3)C2=CC1O	Enamine	Z19647032
Aesculetin	OC1=CC2=C(C=C1O)C=CC(O2)=O	Sigma-Aldrich	246673

References

1. Jenne T, Engelhardt L, Baronaite I, Levy D, Riven I, Malolepszy M, et al. Allosteric control of the bacterial ClpC/ClpP protease and its hijacking by antibacterial peptides. EMBO Journal. 2025;

2. Carroni M, Franke KB, Maurer M, Jäger J, Hantke I, Gloge F, et al. Regulatory coiled-coil domains promote head-to-head assemblies of AAA+ chaperones essential for tunable activity control. eLife. 2017 Nov 22;6.

3. Maurer M, Linder D, Franke KB, Jäger J, Taylor G, Gloge F, et al. Toxic Activation of an AAA+ Protease by the Antibacterial Drug Cyclomarin A. Cell Chemical Biology. 2019 Aug 15;26(8):1169-1179.e4.

4. Azinas S, Wallden K, Katikaridis P, Jenne T, Schahl A, Mogk A, et al. Structure of the central Staphylococcus aureus AAA+ protease MecA/ClpC/ClpP. Communications Biology. 2025 Oct 14;8(1):1467.
